# Urinary and oral microbiota in Polish women: a pilot case-control study of breast cancer

**DOI:** 10.3389/fmicb.2025.1538224

**Published:** 2025-04-22

**Authors:** Anna Marciniak, Marzena Skrzypczak-Zielińska, Oliwia Zakerska-Banaszak, Elżbieta Nowakowska, Anna Kozaczka, Brunon Zemła, Andrzej Szpak, Dariusz Godlewski, Jadwiga Charzewska, Dorothy R. Pathak

**Affiliations:** ^1^Center of Cancer Prevention and Epidemiology OPEN, Poznan, Poland; ^2^Institute of Human Genetics, Polish Academy of Sciences, Poznan, Poland; ^3^Affidea International Cancer Center Poznań, Poznan, Poland; ^4^Department of Nephrology, Transplantation and Internal Medicine, Medical University of Silesia, Katowice, Poland; ^5^Department of Nuclear Medicine and Endocrine Oncology, Maria Skłodowska-Curie Memorial Cancer Centre and Institute of Oncology, Gliwice, Poland; ^6^Witold Chodźko Institute of Rural Medicine, Lublin, Poland; ^7^Department of Nutrition and Nutritional Value of Food, National Institute of Public Health NIH-National Research Institute, Warsaw, Poland; ^8^Department of Epidemiology and Biostatistics, Michigan State University, East Lansing, MI, United States

**Keywords:** breast cancer, next-generation sequencing, oral microbiota, urinary microbiota, functional analysis

## Abstract

**Introduction:**

The human microbiota can be a critical component in the development and progression of various diseases, including cancer. This study aims to investigate the composition of the urinary and oral microbiota in Polish breast cancer (BC) patients relative to healthy controls (HCs) and to predict relevant metabolic pathways of microbiota in studied groups.

**Methods:**

Urine and oral samples from 48 participants, 24 BC cases and 24 HCs, randomly selected from 417 BC cases and 514 HCs, were analyzed using next-generation sequencing of bacterial 16S rRNA gene (V1-V9) and fungal ITS regions, along with bioinformatics tools to identify and compare microbial communities and predict relevant pathways of microbiota in the studied groups.

**Results:**

BC case urine microbiota contained an increased abundance of *Corynebacterium* (5.2-fold, but not significant) and *Gammaproteobacteria* including unknown genus and *Pseudomonas* (1.7- and 1.8-fold) and decreased abundance of *Family XI* (0.3-fold) and *Bifidobacteriaceae* (0.4-fold) compared to HCs. Oral BC microbiota contains higher levels of the bacterial families *P5D1–392*, *Leptotrichiaceae*, and *Pasteurellaceae* (3.3-, 3.3-, and 1.9-fold, respectively), whereas the genera *Cellulosimicrobium*, *Pseudomonas,* and *Pantoea* were significantly less abundant (0.4-, 0.3-, and 0.3-fold, respectively). At the species level, the most differentiating species between BC and HC was uncultured *Pseudomonas* sp. (1.8-fold) in urine and *Pantoea agglomerans* (0.2-fold) in oral microbiota. Fungal composition did not show any significant differences between the groups. Functional analysis based on Phylogenetic Investigation of Communities by Reconstruction of Unobserved States (PICRUSt2) predicted, e.g. enhanced hydrogen production and benzoyl-CoA degradation in BC cases, as well as reduced CMP-diacetamido-8-epilegionaminic acid biosynthesis.

**Discussion:**

The study underscores the potential significance of the microbiota in BC pathogenesis. Further research is needed to elucidate the mechanisms underlying microbiota–tumor interactions and to explore the clinical applications.

## Graphical abstract

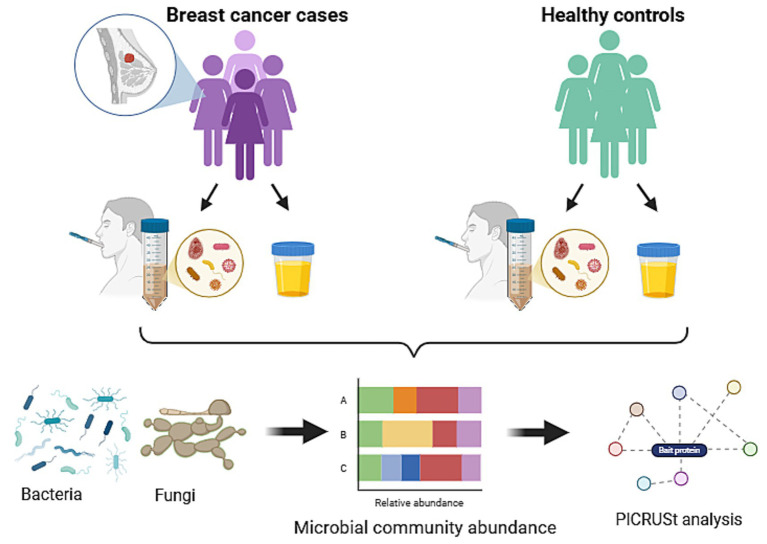

Skrzypczak-zielinska, M. (2025) (https://BioRender.com/o59f811).

## Introduction

Based on the World Health Organization (WHO) data, breast cancer (BC) is the most frequently diagnosed cancer in the world. In 2020, 2.3 million people were diagnosed with BC and 685,000 cases died from this disorder (Sung et al., 2021). It is estimated that by 2040, the number of newly diagnosed BC worldwide will increase by 40% and the number of deaths will escalate by 50% (Arnold et al., 2022). In Poland, the incidence and mortality of BC are a significant health problem. In 2020 represented 23% of all cancer cases, and was the second cause of cancer-related deaths among Polish women (Wojciechowska et al., 2023).

The human body is inhabited by a microbiota consisting mainly of bacteria, fungi, archaea, and viruses (Proctor et al., 2019). The composition and role of the intestinal microbiota is crucial in maintaining the homeostasis of the host organism. It is involved in a variety of physiological processes, including immune system modulation, metabolic homeostasis, and defense against pathogens, with its influence extending to distant organs and potentially impacting cancer development (Álvarez-Mercado et al., 2019, 2023; Vivarelli et al., 2019). In the context of BC, the microbiota may contribute to pathogenesis by regulating steroid hormone levels, energy metabolism, the synthesis of bioactive metabolites (e.g., genotoxins, lipopolysaccharides, and vitamins), and immune response modulation. It has been demonstrated that the bacterial enzyme *β*-glucuronidase (GUS) participates in estrogen reactivation, which may contribute to BC progression (Al-Ansari et al., 2021). Short-chain fatty acids (SCFAs), including acetate, propionate, and butyrate, may support cellular stability and modulate immune responses, potentially exerting protective effects. In contrast, secondary bile acids exhibit both carcinogenic and anti-carcinogenic properties, depending on the biological context and microbiota composition (Miko et al., 2019). The complex interactions between the microbiota and the human organism suggest that its disturbances, known as dysbiosis, may not only contribute to the development of BC but also influence its progression and treatment efficacy (Viswanathan et al., 2023). Research findings demonstrate that tumor tissues exhibit reduced microbial diversity compared to healthy tissues, along with an increased abundance of bacteria from the *Pseudomonadaceae* and *Enterobacteriaceae* families. In contrast, *Propionibacterium* and *Staphylococcus* are more frequently found in healthy tissues, suggesting their potential role in maintaining microenvironmental homeostasis (Tzeng et al., 2021).

For BC, several studies evaluated the presence of various bacteria in the human gut, breast tissue, and oral microbiome (Huybrechts et al., 2020; Samkari et al., 2022; Thu et al., 2023) with inconsistent findings. While Wang et al. (2017). and Nearing et al. (2020) found no differences in the distribution of oral microbiome between BC cases and healthy subjects, Wu et al. (2022) observed a lower abundance of *Porphyromonas* and *Fusobacterium* genus in BC cases.

The urinary microbiota has been less studied; however, recent studies showed its association with oncological disease (Wolfe and Brubaker, 2015; Perez-Carrasco et al., 2021), including bladder, colon, and prostate cancer. For BC, Wang et al. demonstrated increased amounts of Gram-positive bacteria, including *Corynebacterium, Staphylococcus, Actinomyces* and *Propionibacteriaceae* in the urine microbiota of BC cases (Wang et al., 2017).

Moreover, although fungi constitute a minor part of the microbiota community, their association with carcinogenesis has been established. Therefore, their composition and abundance could be important in BC cases. For example, *Candida albicans* can support cancer progression (Ramirez-Garcia et al., 2013).

The current research investigated the composition of urinary and oral microbiota in Polish BC patients relative to healthy subjects, marking Poland’s first study of its kind. The findings may help identify potential biomarkers and offer new prevention or treatment options for BC through microbiota intervention.

## Materials and methods

### Study population

This study is based on a subset of samples collected as part of the Polish Women’s Health Study (PWHS) which was conducted between 2000 and 2006 (Wajszczyk et al., 2021). The biological samples were collected between 2004 and 2006 with participants from five Regional Cancer Registries located in three regions in Poland. The study followed the Helsinki Declaration and received approval from the local Bioethics Committee of the Greater Poland Medical Chamber (resolution no 44/2024 issued on 20 March 2024). BC cases were consecutively included in the study, simultaneously from Regional Registries, starting on 1 January 2000, until the assumed number of cases (200 per register) was exhausted, regardless of the type of breast cancer. Control samples were matched by age and residence. For further details, see Wajszczyk et al. (2021) and Łukasiewicz-Śmietańska et al. (2024). Urine and oral wash samples were collected from 514 healthy women and 417 women with BC under the sub-study “Breast Cancer Gene-Diet Interaction in Polish Women.” This pilot project includes samples from 48 participants—randomly selected 24 women diagnosed with BC and 24 healthy controls equally from each of the five study sites (48 urine samples and 47 oral wash samples) using a simple, dependent sampling method. Participants collected midstream morning urine in 50 mL containers with Vitamin C after fasting for 8 h; menstruating participants were asked to collect morning urine samples between the 20th and 25th day of the cycle. Oral wash samples were collected using 50 mL Falcon tubes and Scope mouthwash after fasting for 1 h. Urine samples and oral washes were transported in a carrier on ice to freezers at study sites, then to Warsaw for storage at −80°C, and finally to Poznań in 2018, where they remain at −80°C.

### DNA extraction

Microbial genomic DNA was extracted using the Sherlock AX kit (A&A Biotechnology, Gdansk, Poland) according to the manufacturer’s protocol. After defrosting each urine sample, 4 mL of sterile 1 M TRIS-EDTA buffer was added, mixed, and centrifuged at 3,645 x g at 4°C for 10 min. Next, the supernatant was discarded. The urine pellet at the volume of 300 μL was lysed with 300 μL of lysis buffer, 20 μL of proteinase K (20 mg/mL), 20 μL of lysozyme (10 mg/mL), 5 μL of mutanolysin (10 U/μL), and 10 μL of lyticase (10 U/1 μL). Oral rinses were directly lysed without concentration. The isolated microbial material was quantified and characterized using a spectrophotometer NanoDrop^™^ 2,000 (Thermo fisher scientific, Inc., Waltham, MA, United States).

### Next-generation sequencing (NGS)

Bacterial 16S ribosomal RNA (rRNA) gene libraries including all nine hypervariable V1-V9 region and fungal ITS region libraries for metaprofiling analysis were prepared using the QIAseq 16S/ITS Panels Reagent Kit Screening Panel (QIAGEN) according to the manufacturer’s protocol. The obtained amplification products were combined, mixed, and then cleaned using the magnetic QIAseq Beads (QIAGEN) and subjected to indexing with unique oligonucleotide sequences placed on a commercial plate QIAseq 16S/ITS 96-Index I (QIAGEN). Amplicon libraries were purified, and the following were checked using a High Sensitivity DNA Assay on an Agilent 2,100 Bioanalyzer System (Agilent, Santa Clara, CA, USA) and pooled to one collective NGS library with a final concentration of 8 pm. The bacteriophage *Φ*-X174 (PhiX) control library was added at 3%. Sequencing was performed on an Illumina MiSeq platform using a MiSeq Reagent Kit v3 (600 cycles).

### Bioinformatics

Sequencing reads (paired-end) of the 16S rRNA gene and the ITS1-ITS2 region were analyzed using the Detect Amplicon Sequence Variants algorithm, based on DADA2 (Callahan et al., 2016), in CLC Genomics Workbench v. 24.02 with CLC Microbial Genomics Module v. 24.1.1 (QIAGEN Bioinformatics, Aarhus, Denmark). The Detect Amplicon Sequence Variants analysis included the following steps: (1) initial error filtering (maximum expected errors per read: 1.0) and length trimming (first/s read length: 250 bp for both reads), (2) dereplication, (3) denoising, (4) removal of chimeric sequences, and (5) merging of unique read pairs (with a minimum overlap of <12 bases). Taxonomic annotation of amplicon sequence variant (ASV) abundance tables was performed using the Assign Taxonomies to Sequences in Abundance Table tool, with a minimum similarity threshold of 80%. ASVs were mapped against the following reference databases: SILVA SSU 99% (release 138.1) for bacterial classification and UNITE 97% (version 9.0) for fungal classification. Bioinformatics processing made it possible to obtain and export Excel files with a qualitative–quantitative list of microorganisms on different taxonomic levels: phylum, class, order, family, genus, and species. Alpha diversity was calculated using the ASV richness. Beta diversity was specified based on a Bray–Curtis dissimilarity matrix. To predict the functional potential of the microbial community of urine and oral cavity microbiota in the BC patients and healthy control groups, the PICRUSt2 (Phylogenetic Investigation of Communities by Reconstruction of Unobserved States) tool integrated within CLC Genomics Workbench v. 24.02 with CLC Microbial Genomics Module v. 24.1.1 (QIAGEN Bioinformatics, Aarhus, Denmark) was used. The analysis was performed based on ASV abundances from study samples using the default prediction model. Prior to functional prediction, ASVs were filtered based on a minimum relative abundance threshold of 0.01%, and normalization was applied to account for 16S rRNA copy number variation. Metabolic pathways were annotated using the MetaCyc database. The differential abundance of pathways was assessed using the Kruskal–Wallis test. Visualization of statistically significant (*p* < 0.05) functional profiles and pathway abundances was performed using heatmaps generated in GraphPad Prism 8 (GraphPad Software, Inc., San Diego, United States) software.

### Statistical analysis

The normality of microbiota percentage abundance levels at different taxonomic levels was assessed using the Shapiro–Wilk test. When the data did not follow the normal distribution pattern, the non-parametric Mann–Whitney test for median values of cases and controls was applied for the comparison. For the relative abundance analyses, we restricted to taxon present in at least 20% of the population. Calculations were carried out using PQStat 1.8.6 (PQStat Software, Poznan. Poland) and Graph Pad Prism 8 (GraphPad Software, Inc., San Diego, United States) software, and all tests were considered significant at a *p*-value of ≤0.05. The FDR-adjusted *p*-values were calculated according to the Benjamini–Hochberg procedure (Benjamini and Hochberg, 1995).

## Results

A total of 48 participants were included in this study: 24 women with BC and 24 HC. The characteristics of the study groups are shown in Table 1 and were reported during the interview. BC cases were similar to healthy controls with regard to all presented parameters (*p* > 0.05).

**Table 1:** Characteristic of the study population.

Parameter continues (mean ± SD) Categorical (*n*, %)	Total, *n* = 48	Breast cancer, *n* = 24	Healthy controls, *n* = 24	*P*-value
Age (years)	60 ± 10.8 (35–83)	60.17 ± 10.95 (43–83)	59.58 ± 10.97 (35–79)	0.85
Postmenopausal	*n =* 45 (93.75%)	*n =* 24 (100%)	*n =* 21 (87.5%)	0.07
Menopause age (years)	48.82 ± 3.92 (39–56)	48.04 ± 4.18 (39–55)	49.81 ± 3.36 (42–56)	0.13
Current smokers	16 (33.3%)	9 (37.5%)	7 (29.2%)	0.54
Current alcohol drinkers	30 (62.5%)	15 (62.5%)	15 (62.5%)	>0.99
BMI*	27.38 ± 4.74 (18.06–40.9)	27.39 ± 5.18 (18.06–38.39)	27.44 ± 4.46 (18.59–40.9)	0.98

*At diagnosis for BC cases and interview for controls, SD, standard deviation.

**Figure 1:**
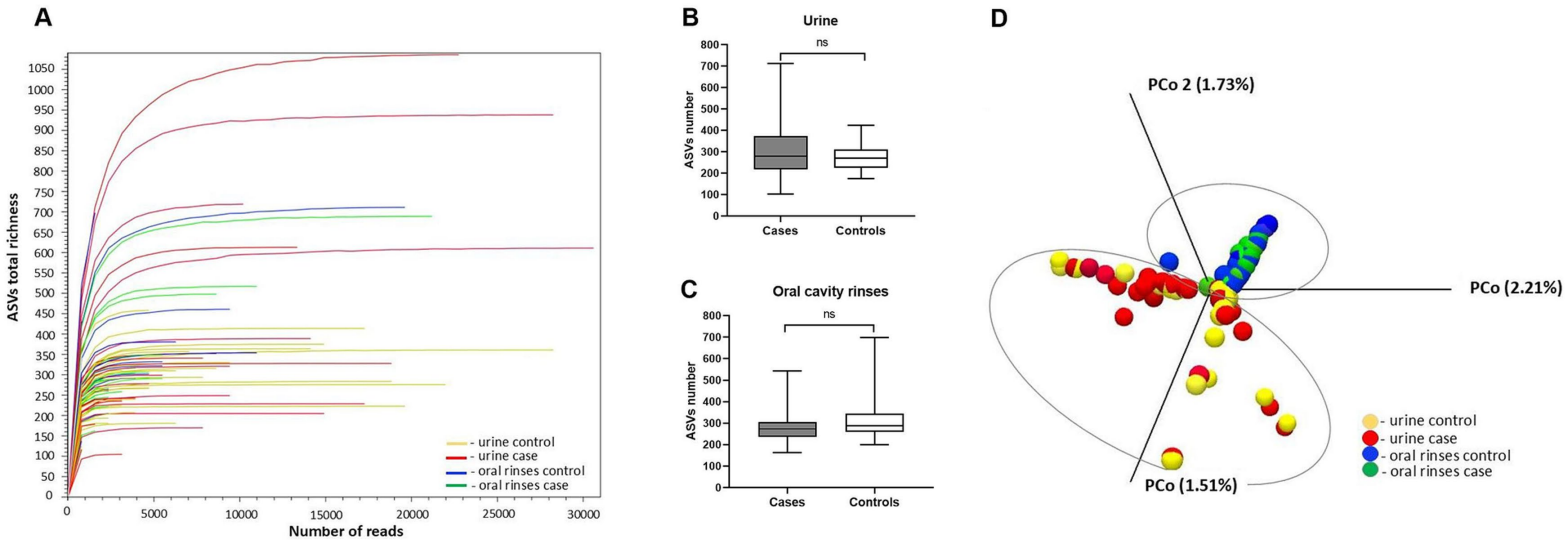
Alpha and beta diversity of the urine and oral cavity rinses microbiota between patients and healthy controls: **(A)** refraction curves of amplicon sequence variant (ASV) richness at read depth in all analyzed samples; **(B)** ASV richness in urine samples; **(C)** ASV richness in oral cavity rinses samples; **(D)** comparison between sample diversities by principal coordinate analysis (PCoA) with a Bray–Curtis dissimilarity matrix.

In the NGS analysis for urine and oral metaprofiling, the obtained parameters were as follows: the flow cell cluster density 1,006 ± 32, the Phred base-calling score GQX ≥ 30, an average of 74.87%, and the passing filter (PF) reads 22,703,315 constituting 92.25% of the total reads 24,597,294. In the bioinformatics analysis, the number of input reads per sample was on average 410,976 [SD 68509.5 (median 402,000, min. 278,462, max. 698,298)], and the number of reads after filtering on length, ambiguity, and expected errors was at the level of 24639.6 (SD 6637, median 24,318, min. 12,764, max. 47,286). Refraction curves of amplicon sequence variant (ASV) richness at read depth are presented in Figure 1A. In our study, both urine and oral rinse samples of BC patients and controls demonstrated similar microbial taxonomic diversity based on the deepest available analysis of all regions V1-V9 of the 16S rRNA gene (Figures 1B,C). Analysis of the beta diversity determined by the Bray–Curtis dissimilarity revealed that the bacterial microbiota of urine samples create a separate cluster from oral microbiota samples (Figure 1D).

**Figure 2:**
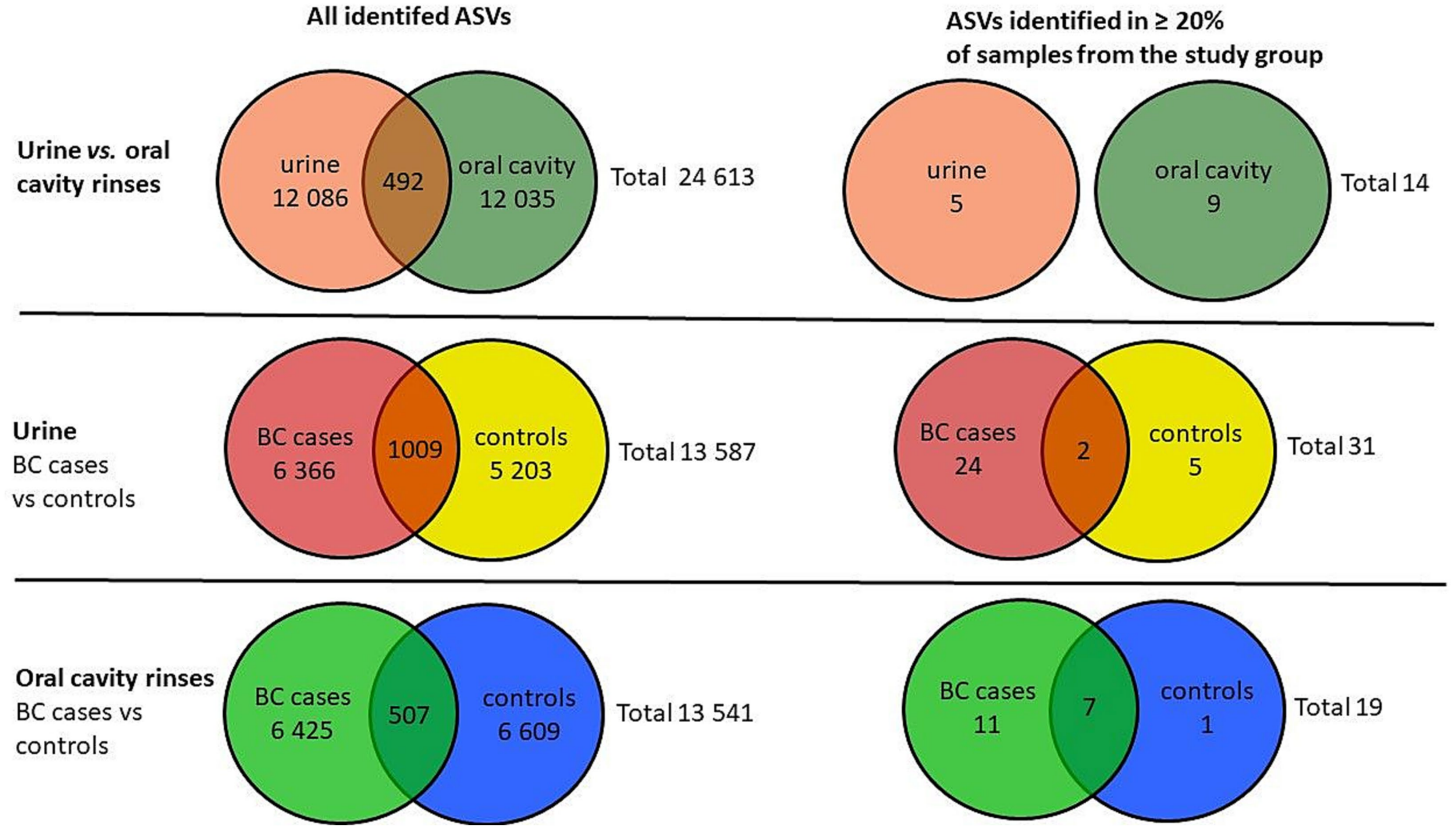
Venn diagrams of the number of identified ASVs in urine and oral cavity samples of BC cases and the control group.

Based on a total number of identified ASVs, the urine samples presented a similar richness of microbiota compared to oral rinses, 12,086 *vs*. 12,035, respectively, of which 492 ASVs are found in both types of samples, giving a total richness of 24,613 ASVs. To narrow down the number of ASVs and to exclude sporadically occurring microorganisms, only ASVs that were observed in at least 20% of the samples within each group were counted (Figure 2).

After applying this criterion, urine samples presented 5 unique ASVs and 9 oral rinses, and none were joint. For urine, BC cases showed more unique ASVs than controls (24 *vs*. 5), with 2 ASVs present in both groups. In oral rinses, BC patients demonstrated also a richer composition of unique ASVs (11) compared to healthy controls (1) (Figure 2).

NGS raw data are available in the SRA database (accession number PRJNA1180501).

### Metagenomic profile of the urine samples

**Figure 3:**
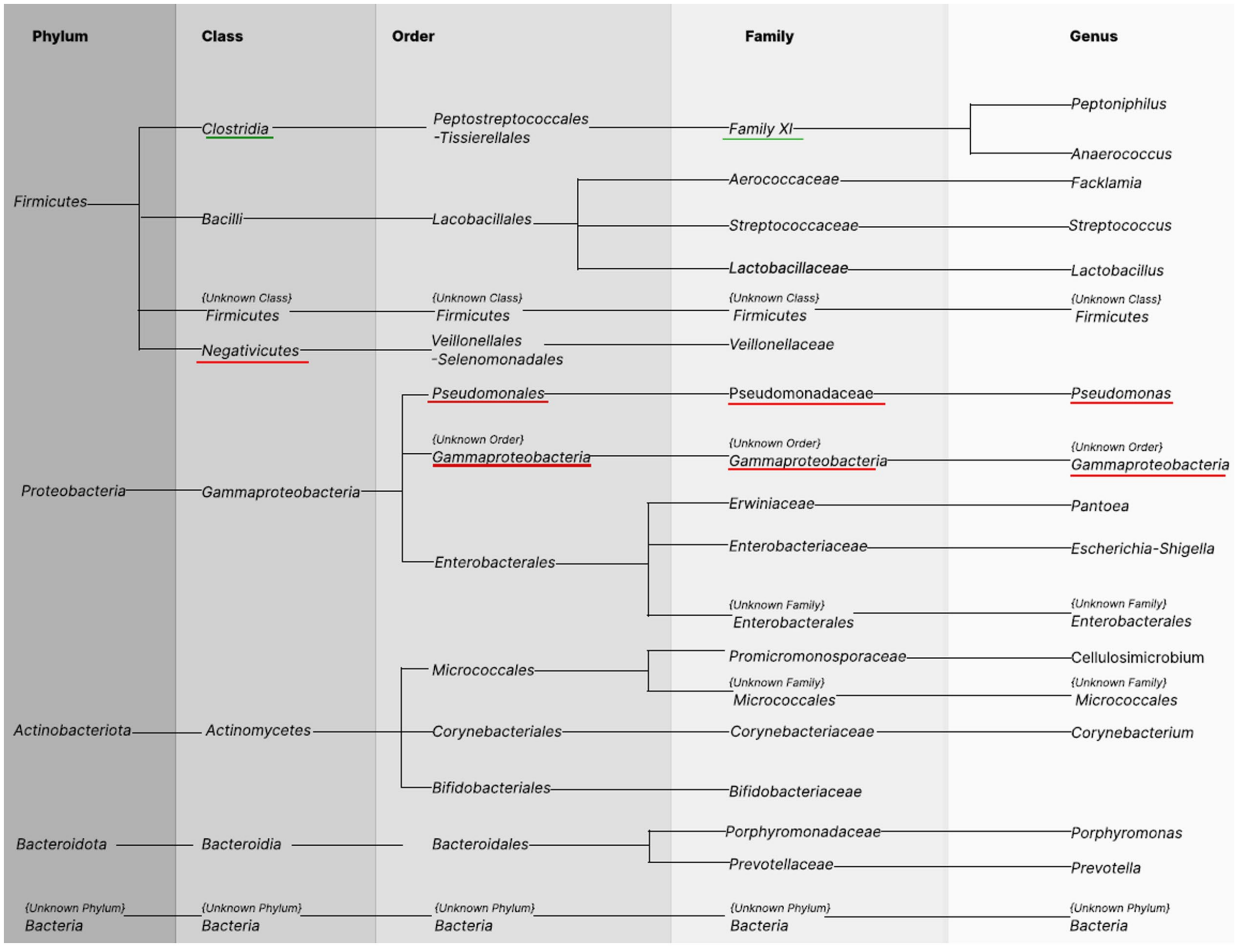
Bacterial taxonomy in the urine samples. Columns show names of taxonomic levels identified in urine samples of BC patients and healthy controls occurring in at least 20% of the samples in the total group. The first column represents the phylum level. The subsequent taxonomy groups are class, order, family, and genus. The green underline indicates greater abundance among healthy controls, and the red underline indicates greater abundance among cases.

We analyzed the bacterial taxonomic composition of urine sample microbiota in both the BC patients and the control group. A total of 13 phyla, 19 classes, 39 orders, 83 families, 159 genera, and 265 species were detected (Supplementary Tables S1–S6). After applying our filter in the biostatistics (selection of taxa occurring in at least 20% of the samples), the number on each taxonomic level was reduced to 5 phyla, 8 classes, 12 orders, 18 families, 17 genera, and 47 species (Figure 3—species not shown). Obtained results are presented in tabular form in the Supplementary Tables S1–S6.

The most abundant bacteria for the whole study group at the phylum level in urine were the following: *Bacillota* (commonly known as *Firmicutes*, 32.66%, SD 37.78), *Pseudomonadota* (known as *Proteobacteria*, 31.16%, SD 29.81), and *Actinomycetota* (known as *Actinobacteriota*, 25.77%, SD 25.44). Bacteria classified at these three phyla accounted for 86.22% of cases and 88.83% of controls. However, the composition of urine microbiota at the phylum level did not differ significantly statistically between BC cases and HCs.

**Figure 4:**
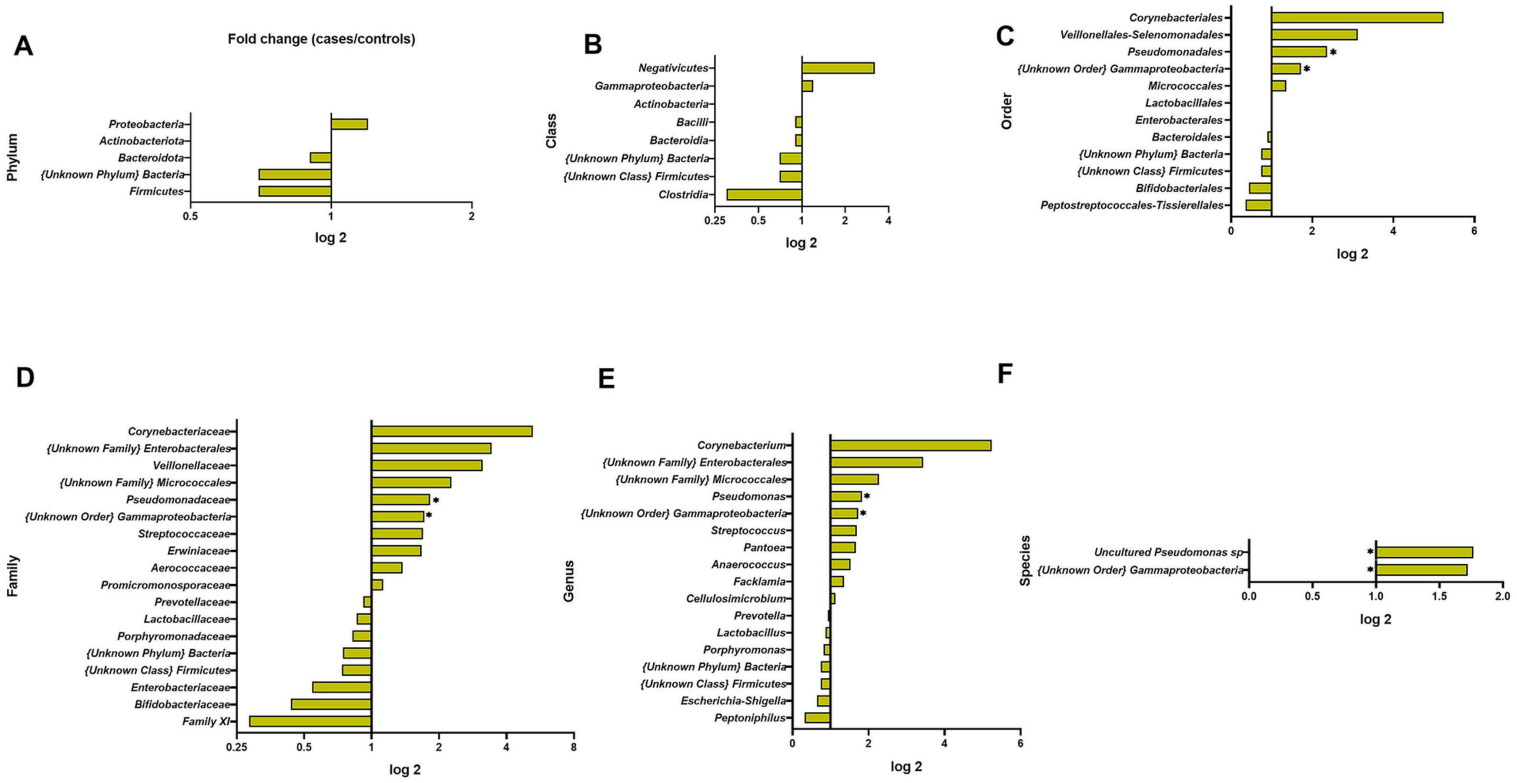
Fold changes of urine bacterial abundance between cases and healthy controls at different taxonomic levels: **(A)** phylum; **(B)** class; **(C)** order; **(D)** family; **(E)** genus; **(F)** species. * indicates statistical significance at *p* ≤ 0.05, before FDR correction. ** *p* ≤ 0.01.

Differences in abundance between BC cases and HCs were observed at further taxonomic classification levels (Figures 3, 4). For the results discussed below, we provide *p*-values (*p* ≤ 0.05), for the difference in bacterial abundance between BC cases and HCs on the Mann–Whitney test, before applying FDR correction. Both sets of p-values are reported in Supplementary Tables S1–S6.

At the bacterial class level, the results showed a lower abundance of *Clostridia* in BC patients compared to HCs (0.3-fold) and a higher level of *Negativicutes* (3.2-fold); these differences were not statistically significant (Figure 4).

At the bacterial order level, the most abundant in the whole study group were *Lactobacillales* (26.53%, SD 35.48), *Enterobacterales* (20.21%, SD 26.21), and *Micrococcales* (17.33%, SD 20.72) and did not differ significantly between BC cases and HCs. In BC samples, we observed a significantly higher presence of *Pseudomonadales* (2.4-fold) and {Unknown Order} *Gammaproteobacteria* (1.7-fold) (*p* = 0.0077, *p* = 0.0424, respectively).

At the family level, the urine samples of BC cases and HCs were dominated by *Lactobacillaceae* (18.56%, SD 32.65), *Promicromonosporaceae* (13.09%, SD 16.05), and *Enterobacteriaceae* (11.04%, SD 27.24) and did not differ between BC cases and HCs. BC cases had a 1.8-fold and 1.7-fold significantly higher abundance of *Pseudomonadaceae* (*p* = 0.0257) and Unknown Order} *Gammaproteobacteria* (*p* = 0.0424) (Figure 4).

At the genus level, we observed 17 types of bacteria, of which *Lactobacillus* (17.50%, SD 31.28) and *Cellulosimicrobium* (12.82%, SD 15.99) predominated among urine samples and did not differ between BC cases and HCs. However, the less common genera of the microbiota composition differentiated between BC patients and HCs: *Pseudomonas* and {Unknown Order} *Gammaproteobacteria* were significantly more common in BC (1.8-fold, *p* = 0.0257, 1.7-fold *p* = 0.0424, respectively, Figure 4).

At the species level, a significant difference revealed the uncultured *Pseudomonas* sp. (31.8-fold, *p* = 0.028) and *Gammaproteobacteria* {unknown order} (1.7-fold, *p* = 0.042) in urine with greater abundance among BC patients (Figure 4).

Fungal analysis revealed the presence of two phyla, *Ascomycota* and *Basidiomycota* in urine material, detected in 16.6% of samples (8 of 48). At lower taxonomic levels, urine samples represented 8 classes, 13 orders, 17 families, 19 genera, and 18 species (detailed in Supplementary Table S13, respectively). The occurrence of these taxa was sporadic, typically limited to individual samples. No differences in urine fungal composition were observed between BC patients and controls. The only notable distinction was a higher abundance of the phylum *Basidiomycota* (*p* = 0.0392), class *Agaricomycetes* (*p* = 0.0392), order *Capnodiales* (*p* = 0.0192), and family *Cladosporiaceae* (*p* = 0.0192) in the urine of BC patients compared to controls. However, this difference was no longer significant after adjustment.

### Metagenomic profile of oral rinse samples

**Figure 5:**
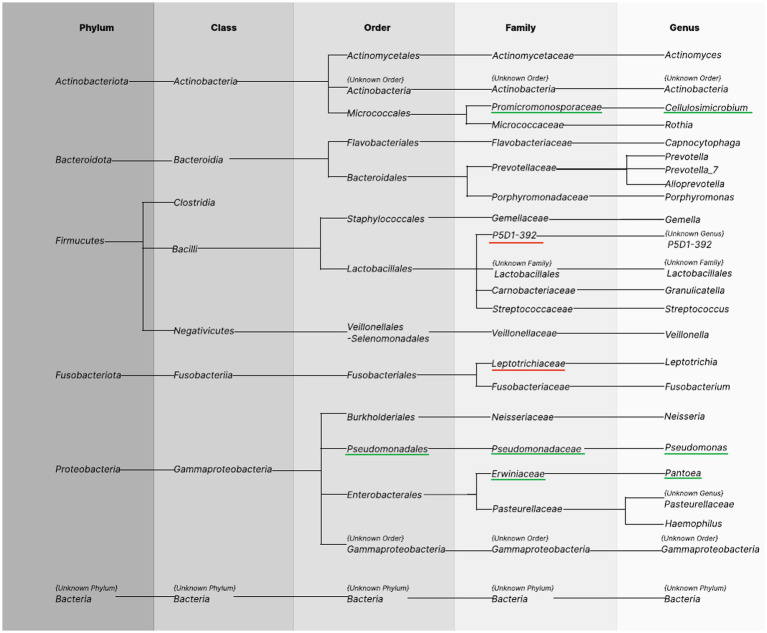
Bacterial taxonomy in the oral rinse samples. Columns show phyla identified in oral rinse samples of BC patients and healthy controls occurring in at least 20% of the samples in the total group. The first column represents the phylum level. The subsequent taxonomy groups are class, order, family, and genus. The green underline indicates greater abundance among healthy controls, the red underline indicates greater abundance among cases.

A total of 9 phyla, 15 classes, 45 orders, 53 families, 80 genera, and 240 species of bacteria were detected, wherein after applying our filter in the biostatistics (selection of taxa occurring in at least 20% of the samples), the number on each taxonomic level was reduced to 6 phyla, 8 classes, 14 orders, 21 families, 24 genera, and 44 species (Figure 5—species not shown). All findings are presented in tabular format within the Supplementary Tables S7–S12.

**Figure 6:**
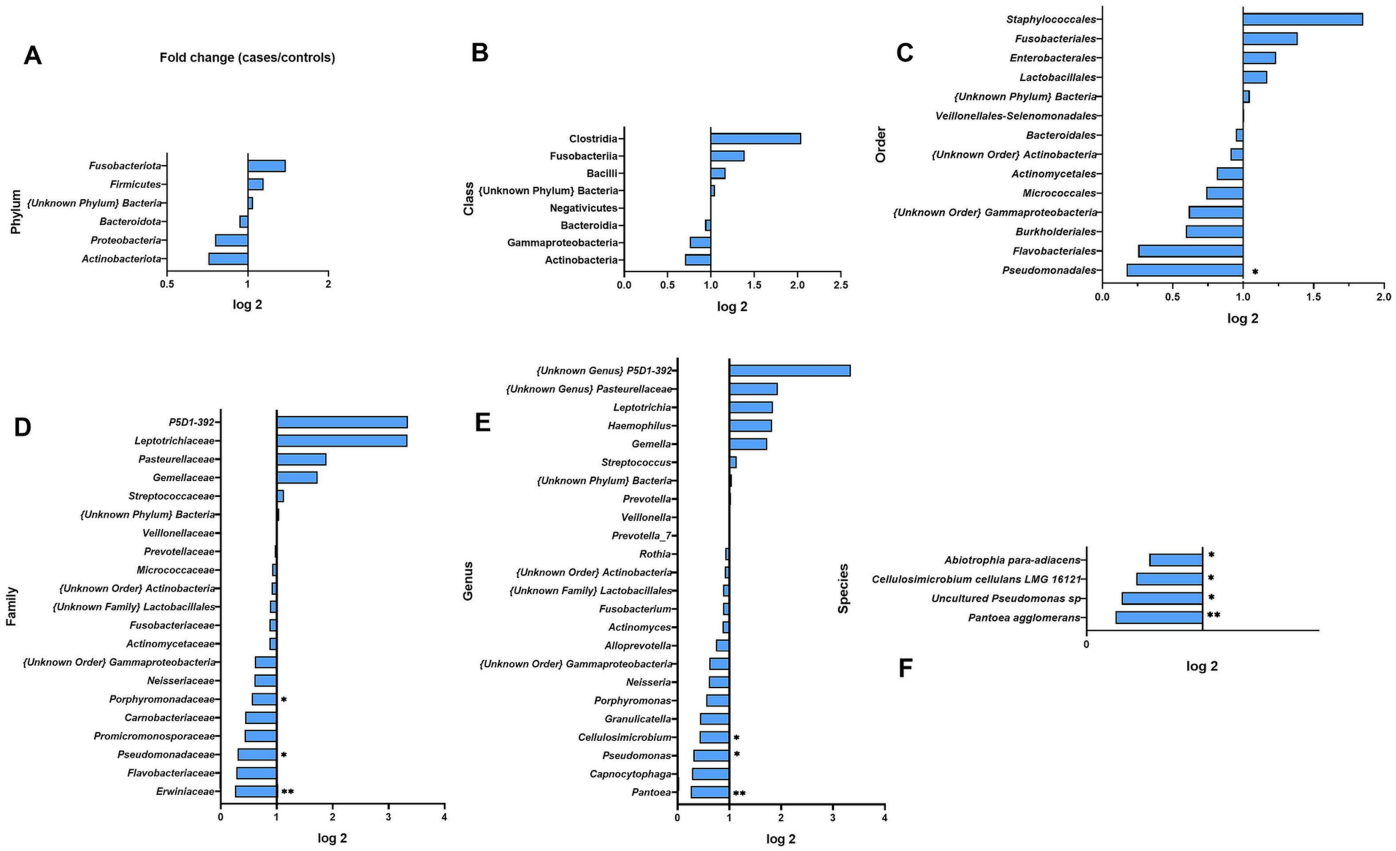
Fold changes of oral wash bacterial abundance between cases and healthy controls at different taxonomic levels: **(A)** phylum; **(B)** class; **(C)** order; **(D)** family; **(E)** genus; **(F)** species. * indicates statistical significance at *p* ≤ 0.05, before FDR correction. ** *p* ≤ 0.01.

At the phylum level, *Firmicutes* (51.2%, SD 26.4) and *Bacteroidota* (30.5%, SD 23.6) were most abundant in both BC cases and HCs accounted for 84.04% of all bacteria present in cases and 79.28% in controls. The distribution of bacterial phyla did not reveal statistically significant differences between BC cases and healthy controls (Figure 6).

At the class level, the most abundant ones were *Bacilli* (46.47%, SD 29.81 in BC and 39.69%, SD 28.12 in HCs) and *Bacteroidia* (29.39%, SD 23.64 in BC and 31.53%, SD 37.85 in HC); however, significant differences were not observed between groups.

At the order level, the most abundant were *Lactobacillales* (42.44%, SD 28.34) and *Bacteroidales* (30.5%, SD 22.59), which accounted for 75.01% in BC patients and 69.95% in controls. Only *Pseudomonadales* had significantly lower levels in BC cases relative to HCs (0.2-fold, *p* = 0.0203).

At the family level, the oral rinse samples of both groups were dominated by *Streptococcaceae* (35.87%, SD 26.66) and *Prevotellaceae* (29.01%, SD 22.77). We observed a 0.3-fold, 0.4-fold, and 0.3-fold lower abundance of *Erwiniaceae*, *Promicromonosporaceae*, and P*seudomonadaceae* among BC cases (*p* = 0.0091, *p* = 0.0332, and *p* = 0.0470) (Figure 6). We observed a higher richness among BC cases of *Leptotrichiaceae* (3.3-fold) and P5D1–392 family (3.3-fold) but not statistically significant.

At the genus level, we observed 24 representatives of bacteria, and the most numerous were *Streptococcus* (35.66%, SD 26.42) and *Prevotella_7* (23.74%, SD 22.04). Genera of *Pantoea, Cellulosimicrobium, and Pseudomonas* were less abundant in BC patients (0.3-, 0.4-, and 0.3-fold, *p* = 0.0091, *p* = 0.0332, and *p* = 0.0470 respectively).

At the species level, four species differed significantly between BC cases and HCs. As shown in Figure 6 and Supplementary Table S12 all of those species, *Abiotrophia para-adiacens*, *Cellulosimicrobium cellulans* LMG 16121, *Pseudomonas* sp. (uncultured), and *Pantoea agglomerans* were less abundant among BC cases (0.2-, 0.4-, 0.3-, and 0.5-fold; *p* = 0.0074, *p* = 0.0332, *p* = 0.0470, and *p* = 0.0476).

Fungal analysis revealed in oral cavity material the presence of two phyla, *Ascomycota* and *Basidiomycota*, detected in 19.14% of samples (9 of 47). At lower taxonomic levels, oral cavity samples represented 8 classes, 13 orders, 17 families, 19 genera, and 18 species (detailed in Supplementary Table S14). The occurrence of these taxa was sporadic, typically limited to individual samples. No differences in oral cavity fungal composition were observed between BC patients and controls.

### Functional profile analysis of the urine and oral cavity microbiota

**Figure 7:**
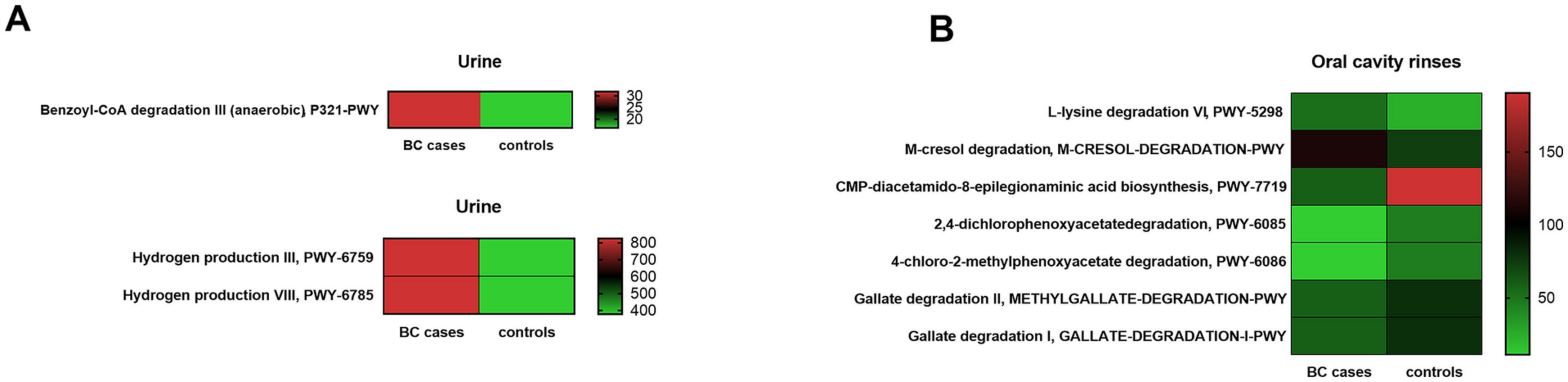
Functional pathways predicted by PICRUSt2 differentiate BC cases and healthy control (HC) groups in urine **(A)** and oral rinses **(B)**. Differentiating MetaCyc pathways identified by Kruskal–Wallis test (*p* < 0.01). The color scale (red means higher and green means lower) corresponds to the raw abundance, based on the number of reads. The column on the right side corresponds to the number of sequencing reads.

Using PICRUSt2 software, based on inferred Enzyme Commission (EC) profiles, we identified 1,352 predicted pathways in urine samples and 1,323 in oral rinses. Among them, three metabolites from urine and seven from oral rinses were identified as most significantly differentiating between BC patients and healthy controls (*p* < 0.05, Kruskal–Wallis test). These are presented in Figure 7A and Supplementary Table S15 for the urine metabolome, and in Figure 7B and Supplementary Table S16 for the oral metabolome.

Prediction analysis in our urine microbiota data found a significantly higher content of enzymes responsible for benzoyl-CoA degradation and hydrogen production in BC cases. A detailed list of predicted urine metabolome enzymes with pathway description information is provided in Supplementary Table S15.

Based on oral microbiota data, we observed a significant predomination of enzymes, such as involved in L-lysine degradation and m-cresol degradation for BC cases, whereas significant reduction in the BC group compared to the control was observed for CMP-diacetamido-8-epilegionaminic acid biosynthesis, 2,4-dichlorophenoxyacetate degradation, 4-chloro-2-methylphenoxyacetate degradation, methylgallate, and gallate degradation. A detailed list of predicted oral rinse metabolome enzymes with pathway description information is provided in Supplementary Table S16.

## Discussion

In our study, metagenomic profiling based on next-generation sequencing allowed us to classify bacteria and fungi inhabiting the urine and oral cavity of BC patients and healthy participants.

Despite contamination of urine samples by bacteria originating from the uroepithelium, urethra, or genital tract, analyzing urine microbiota can provide valuable health insights into its role in disease development (Gottschick et al., 2017; Perez-Carrasco et al., 2021). Previous research on the urine microbiota indicated its lower richness compared to the number of bacteria inhabiting other niches of the human body (Pearce et al., 2014), a finding confirmed in our study, where urine samples had fewer ASVs than oral rinse samples. This is probably the result of greater external environmental pressure in the case of oral wash samples compared to urine samples.

We observed a slight increase in abundance (not significant statistically) of *Proteobacteria* phylum in the group of BC patients (34.5%) compared to healthy controls (27.7%) (Figure 4A). This phylum includes a wide variety of potentially pathogenic genera *Escherichia*, *Klebsiella*, and *Shigella,* whereas *Firmicutes* were presented decreased in BC cases to healthy controls, with an abundance of 25.8 and 35.3%, respectively. In general, the *Proteobacteria* increase with *Firmicutes* depletion is a microbial signature of dysbiosis in gut microbiota (Kumar and Kumar, 2022). Furthermore, in urine BC samples, we detected a significant increase in the genera *Pseudomonas* (*p* = 0.0257) and *Gammaproteobacteria* (*p* = 0.0424) compared to controls. This was also observed at the species level, although the fold change was not so high (Figures 4E,F). There are reports that some strains of bacteria, including *Pseudomonas*, produce toxins that can affect host cells, damaging their DNA and causing genetic changes that promote the development of cancer. On the other hand, there is evidence that cancer cells can stimulate the secretion of toxins by bacteria, such as those from the *Pseudomonas* genus (Choi et al., 2023). When considering the potential link between *Gammaproteobacteria* and BC, we came across research showing that tumor-adjacent-normal tissue was found to contain higher relative abundances of *Gammaproteobacteria* (unclassified) in comparison with healthy normal tissue (Giallourou et al., 2021). The exact role of this bacteria in BC development is still unclear; however, it is known that *Gammaproteobacteria* is associated with a specific lipid profile.

The highest fold change (5.2-fold) with a predominance in BC was presented for *Corynebacterium.* Although most species of *Corynebacterium* are symbiotic to the host, some of them may become pathogenic and lead to infections (Burkovski, 2018). Importantly, in urine samples of healthy women, we observed a higher content of probiotic bacteria from the *Bifidobacteriaceae* and *Lactobacillaceae* families compared to BC. Moreover, BC patients had an increased abundance of *Micrococcales* in their urine. *Micrococcales* belongs to the *Actinomycetes* class, which in general are commensal organisms and can also be an opportunistic pathogen, particularly in a host with an impaired immune system (Li et al., 2022). The presence of the *Micrococcales* bacteria has also been reported in a study analyzing microbiota composition in BC and healthy tissue in Slovakian and Chinese women (Hadzega et al., 2021). In that study, a higher level of *Micrococcales* was observed in the breast tumor tissue of Slovakian women in comparison with healthy breast tissue samples but not for the Chinese women. The observed enrichment of *Micrococcales* in the breast tissue among the Slovakian BC cases and in urine among Polish BC cases, two similar ethnic groups, makes this bacterium a possible candidate as a biomarker for BC in the Polish population.

At this moment, it is difficult to clearly interpret the role of bacteria that significantly distinguish BC patients from healthy women, calling them radical pathobionts or commensals. As defined by the International Cancer Microbiome Consortium, there is a large and poor-defined space between a “pathogen” coexisting with the disease of the host (e.g., *Helicobacter pylori*) and the “commensal” which may contribute to disease under certain conditions. Therefore, the term “dynamic symbiont” was introduced and is often used, also in our study, when interpreting the bacterial genera, such as *Micrococcales*, *Pseudomonas,* and *Gammaproteobacteria* which were enriched in the urine of BC women (Scott et al., 2019).

Searching in our study for the microbial signature of BC in oral rinses, we have observed a significant depletion of bacterial species *Abiotrophia para-adiacens*, *Cellulosimicrobium cellulans LMG 16121*, *Pseudomonas* sp. (uncultured), and *Pantoea agglomerans* in BC cases compared to healthy controls.

To discover the potential functional role of bacteria in the host, we applied metabolomic analysis by PICRUSt2 software to predict relevant pathways of urine and oral cavity microbiota in BC patients and HC. Of the 1,352 identified pathways based on interfered Enzyme Commission (EC) profiles in urine samples and 1,323 in oral rinses, 10 pathways together from both niches differentially enriched between the BC patients and HCs were indicated (Supplementary Tables S15, S16). Based on the urine microbiota composition, we have identified three processes that mostly differentiate the two studied groups, benzoyl-CoA degradation III and hydrogen production III and VIII, which dominate in BC cases. Urinary hydrogen peroxide was postulated to be a biomarker of oxidative stress in general malignancies (Banerjee et al., 2003), whereas increased benzoyl-CoA degradation III (anaerobic) in urine samples of BC patients could be the effect of different factors. Benzoyl-CoA is the most common central intermediate of anaerobic aromatic metabolism, including those aromatic compounds derived from diet, drugs such as chemotherapy, or tumor metabolism (Koch et al., 1993). However, based on the oral rinse microbiota, we have observed that m-cresol and L-lysine degradation was significantly increased in BC cases. There are speculations that cancers alter aromatic compound metabolism leading to an increased degradation process of m-cresol, but there is a lack of evidence. Studies on full microbial metabolites are needed to elucidate these process correlation with BC development.

Fungi were remarkably less present in the tested samples of both urine and oral rinses in our study. In general, we have detected two main fungal phyla, *Ascomycota* and *Basidiomycota,* and also *Incertae sedis* phylum. In the case of oral rinses, we did not find any relevant differences in the composition of mycobiota between patients and healthy women. However, urine samples of BC cases showed a significant reduction in *Basidiomycota* abundance and enrichment in *Agaricomycetes*, *Capnodiales*, *Cladosporiaceae,* and *Cladosporium* abundance, whereas global investigations revealed a strong correlation of particular fungi presence with cancer development (Hosseini et al., 2022). Among them, *Aspergillus*, *Candida*, *Coccidioides*, *Cunninghamella*, *Geotrichum*, *Pleistophora*, *Rhodotorula*, *Filobasidiella*, *Mucor*, *Trichophyton*, *Epidermophyton*, *Fonsecaea*, *Pseudallescheria*, *Penicillium*, *Ajellomyces*, *Alternaria*, *Rhizomucor*, *Piedraia*, and *Malassezia* were found to be linked with breast cancer development (Banerjee et al., 2018). However, these observations concern microbial niches such as intestines or cancer tissue. In our study, we were focused on microbial composition in oral rinses and urine only.

To summarize, the current study has shown bacterial differences in the urinal and oral microbiota composition between BC patients and healthy Polish women. Although the identified differences in bacterial abundance did not maintain statistical significance after FDR adjustment, they suggest trends for further analysis using the full PWHS dataset. We acknowledge the pilot study’s small sample size limits the statistical power. The obtained results will guide us in larger investigations, in which we plan to perform metabolomic analysis parallel to metagenomics profiling in the full sample of 514 HCs and 417 BC cases. Metabolomics data would be a valuable complement to our research, particularly due to estrobolome characterization.

Our study represents the first attempt to compare groups of women that do not differ on age, menopausal status, smoking and drinking habits, and BMI. In previous studies on the diversity of urine and/or oral microbiota in patients with BC conducted by Wu et al. (2022)and Wang et al. (2017), significant differences in the selection of the study and control groups were observed on age, menopausal status, mean BMI, alcohol, tobacco, and antibiotics intake, which authors indicate could have influenced their results. In a research by Nearing et al., although has a fairly extensive description of the study population available, there is a lack of data regarding menopausal status (Nearing et al., 2020). By controlling for age, menopausal status, BMI, smoking, and alcohol consumption, we minimized their impact, allowing us to detect subtle microbiota changes. These findings may have significant implications for the health of women in Poland, suggesting potential new directions for research into BC prevention, diagnostics, and treatment.

## Data Availability

The original contributions presented in the study are publicly available. This data can be found at: http://www.ncbi.nlm.nih.gov/bioproject/1180501.
