# Supplementary material for: Urinary and oral microbiota in Polish women: a pilot case-control study of breast cancer
Source: Front Microbiol. 2025 Apr 22;16:1538224. doi: 10.3389/fmicb.2025.1538224 (PMC12054997; doi:10.3389/fmicb.2025.1538224)
Supplement: Supplementary file 1 [file Table_1.docx]

Supplementary Material

**Table S1.** The percentage abundance of the urine bacteria in the study group of BC cases and controls at the phylum level.

| ***Phylum*** | **BC cases, n = 24** | | | | | **Controls, n = 24** | | | | | ***p*-value** | ***p*-adj.** |
| --- | --- | --- | --- | --- | --- | --- | --- | --- | --- | --- | --- | --- |
|  | **Mean** | **Median** | **SD** | **Min.** | **Max.** | **Mean** | **Median** | **SD** | **Min.** | **Max.** |  |  |
| ***Proteobacteria*** | 34.5603 | 32.2537 | 28.8715 | .0000 | 91.0107 | 27.7604 | 15.6849 | 32.2074 | .0000 | 92.7417 | .2817 | .9444 |
| ***Firmicutes*** | 25.8609 | 8.1272 | 33.0951 | .0000 | 97.3314 | 35.3277 | 11.1167 | 39.5498 | .0000 | 99.9800 | .5463 | .9444 |
| ***Actinobacteriota*** | 25.7961 | 21.8747 | 21.1584 | .0000 | 67.4799 | 25.7456 | 12.8844 | 28.7005 | .0100 | 93.6566 | .7991 | .9444 |
| ***Bacteroidota*** | 7.4543 | .0000 | 18.5676 | .0000 | 77.1845 | 8.2433 | .0070 | 23.1836 | .0000 | 89.2733 | .9035 | .9444 |
| ***{Unknown Phylum} Bacteria*** | 1.2248 | .0000 | 3.2584 | .0000 | 13.6031 | 1.6482 | .0000 | 3.6039 | .0000 | 11.1970 | .9444 | .9444 |

SD - standard deviation; p-value – calculated with the non-parametric Mann–Whitney test or parametric t-test depending on the data normality assessment result; p-adj. - *p* values adjusted with Benjamini-Hochberg false-discovery rate (FDR) for multiple comparisons

**Table S2.** The percentage abundance of the urine bacteria in the study group BC cases and healthy controls at the class level.

| ***Class*** | **BC cases, n = 24** | | | | | **Controls, n = 24** | | | | | ***p*-value** | ***p*-adj.** |
| --- | --- | --- | --- | --- | --- | --- | --- | --- | --- | --- | --- | --- |
|  | **Mean** | **Median** | **SD** | **Min.** | **Max.** | **Mean** | **Median** | **SD** | **Min.** | **Max.** |  |  |
| ***Gammaproteobacteria*** | 33.4790 | 31.7017 | 28.4138 | .0000 | 91.0107 | 27.7604 | 15.6849 | 32.2074 | .0000 | 92.7417 | .3203 | .9444 |
| ***Clostridia*** | 1.1930 | .0000 | 2.1217 | .0000 | 7.8108 | 4.2576 | .0688 | 9.1167 | .0000 | 38.4744 | .3446 | .9444 |
| ***Negativicutes*** | .8529 | .0000 | 3.5175 | .0000 | 17.2915 | .2640 | .0000 | 1.1755 | .0000 | 5.7781 | .6841 | .9444 |
| ***{Unknown Class} Firmicutes*** | 1.1658 | .0000 | 3.2816 | .0000 | 15.8610 | 1.5847 | .0000 | 3.6496 | .0000 | 13.6523 | .7108 | .9444 |
| ***Actinobacteria*** | 25.6416 | 21.4447 | 2.9432 | .0000 | 64.7797 | 25.6696 | 12.8844 | 28.7546 | .0100 | 93.6566 | .8149 | .9444 |
| ***Bacilli*** | 26.7742 | 4.0119 | 35.0879 | .0000 | 94.9662 | 29.2213 | 3.6523 | 37.4491 | .0000 | 99.8623 | .9026 | .9444 |
| ***Bacteroidia*** | 7.4543 | .0000 | 18.5676 | .0000 | 77.1845 | 8.2433 | .0070 | 23.1836 | .0000 | 89.2733 | .9035 | .9444 |
| ***{Unknown Phylum} Bacteria*** | 1.2248 | .0000 | 3.2584 | .0000 | 13.6031 | 1.6482 | .0000 | 3.6039 | .0000 | 11.1970 | .9444 | .9444 |

SD - standard deviation; p-value – calculated with the non-parametric Mann–Whitney test or parametric t-test depending on the data normality assessment result; p-adj. - p values adjusted with Benjamini-Hochberg FDR for multiple comparisons;

**Table S3**. The percentage abundance of the urine bacteria in the study group BC cases and healthy controls at the order level.

| ***Order*** | **BC cases, n = 24** | | | | | **Controls, n = 24** | | | | | ***p*-value** | ***p*-adj.** |
| --- | --- | --- | --- | --- | --- | --- | --- | --- | --- | --- | --- | --- |
|  | **Mean** | **Median** | **SD** | **Min.** | **Max.** | **Mean** | **Median** | **SD** | **Min.** | **Max.** |  |  |
| ***Pseudomonadales*** | 3.3923 | 2.9514 | 3.2253 | .0000 | 12.5058 | 1.4388 | .0369 | 2.0860 | .0000 | 6.6240 | .0077 | .0921 |
| ***{Unknown Order} Gammaproteobacteria*** | 1.0947 | 7.7146 | 1.2223 | .0000 | 38.2095 | 5.8664 | 1.5946 | 7.4902 | .0000 | 25.8348 | .0424 | .2544 |
| ***Micrococcales*** | 19.9618 | 14.5966 | 19.8204 | .0000 | 64.7797 | 14.7034 | 3.2959 | 21.6753 | .0000 | 68.2502 | .1413 | .5651 |
| ***Peptostreptococcales-Tissierellales*** | 1.1472 | .0000 | 2.1401 | .0000 | 7.8108 | 3.2369 | .0688 | 8.3754 | .0000 | 38.4744 | .2449 | .7346 |
| ***Enterobacterales*** | 19.9617 | 15.4711 | 22.5215 | .0000 | 85.0727 | 2.4552 | 6.6206 | 29.9354 | .0000 | 88.9254 | .3842 | .9220 |
| ***Bifidobacteriales*** | 3.9439 | .0000 | 12.4366 | .0000 | 55.8261 | 9.0424 | .0000 | 23.8888 | .0000 | 91.9528 | .4821 | .9641 |
| ***Veillonellales-Selenomonadales*** | .8237 | .0000 | 3.5178 | .0000 | 17.2915 | .2640 | .0000 | 1.1755 | .0000 | 5.7781 | .6841 | .9982 |
| ***{Unknown Class} Firmicutes*** | 1.1658 | .0000 | 3.2816 | .0000 | 15.8610 | 1.5847 | .0000 | 3.6496 | .0000 | 13.6523 | .7108 | .9982 |
| ***Bacteroidales*** | 7.4005 | .0000 | 18.5851 | .0000 | 77.1845 | 8.2433 | .0070 | 23.1836 | .0000 | 89.2733 | .8687 | .9982 |
| ***Lactobacillales*** | 26.7506 | 3.9196 | 35.0911 | .0000 | 94.9662 | 26.3159 | 3.6523 | 36.6121 | .0000 | 99.8623 | .9106 | .9982 |
| ***{Unknown Phylum} Bacteria*** | 1.2248 | .0000 | 3.2584 | .0000 | 13.6031 | 1.6482 | .0000 | 3.6039 | .0000 | 11.1970 | .9444 | .9982 |
| ***Corynebacteriales*** | 1.7226 | .0000 | 7.6354 | .0000 | 37.5155 | .3287 | .0000 | .8709 | .0000 | 3.8961 | .9982 | .9982 |

SD - standard deviation; p-value – calculated with the non-parametric Mann–Whitney test or parametric t-test depending on the data normality assessment result; p-adj. - p values adjusted with Benjamini-Hochberg FDR for multiple comparisons; red font - statistically significant results (*p* < .05)

**Table S4.** The percentage abundance of the urine bacteria in the study group of BC cases and healthy controls at the family level.

| ***Family*** | **BC cases, n = 24** | | | | | **Controls, n = 24** | | | | | ***p*-value** | ***p*-adj.** |
| --- | --- | --- | --- | --- | --- | --- | --- | --- | --- | --- | --- | --- |
|  | **Mean** | **Median** | **SD** | **Min.** | **Max.** | **Mean** | **Median** | **SD** | **Min.** | **Max.** |  |  |
| ***Pseudomonadaceae*** | 2.6119 | 2.4006 | 2.3761 | .0000 | 7.9871 | 1.4330 | .0183 | 2.0900 | .0000 | 6.6240 | .0257 | .3815 |
| ***{Unknown Order} Gammaproteobacteria*** | 1.0947 | 7.7146 | 1.2223 | .0000 | 38.2095 | 5.8664 | 1.5946 | 7.4902 | .0000 | 25.8348 | .0424 | .3815 |
| ***{Unknown Family} Enterobacterales*** | 4.2010 | 1.3059 | 7.5727 | .0000 | 35.9040 | 1.2249 | .8485 | 1.5469 | .0000 | 5.6381 | .0796 | .4175 |
| ***Erwiniaceae*** | 7.9640 | 8.1595 | 7.5240 | .0000 | 23.7858 | 4.7782 | .3723 | 7.1788 | .0000 | 23.0149 | .0928 | .4175 |
| ***Promicromonosporaceae*** | 13.8537 | 1.6936 | 13.2208 | .0000 | 41.6390 | 12.3178 | 1.4256 | 18.7140 | .0000 | 52.6102 | .1590 | .5206 |
| ***{Unknown Family} Micrococcales*** | 4.1110 | .3287 | 6.7357 | .0000 | 23.1407 | 1.8084 | .0053 | 3.7537 | .0000 | 15.6400 | .1825 | .5206 |
| ***Family XI*** | .9125 | .0000 | 1.9143 | .0000 | 7.8108 | 3.2124 | .0688 | 8.2682 | .0000 | 37.8877 | .2025 | .5206 |
| ***Streptococcaceae*** | 3.9769 | .0000 | 16.4053 | .0000 | 8.5069 | 2.3496 | .0000 | 8.5135 | .0000 | 4.9091 | .4098 | .8677 |
| ***Porphyromonadaceae*** | 1.2494 | .0000 | 5.7635 | .0000 | 28.2752 | 1.5248 | .0000 | 4.2192 | .0000 | 16.1628 | .4425 | .8677 |
| ***Bifidobacteriaceae*** | 3.9439 | .0000 | 12.4366 | .0000 | 55.8261 | 9.0424 | .0000 | 23.8888 | .0000 | 91.9528 | .4821 | .8677 |
| ***Enterobacteriaceae*** | 7.7726 | .0000 | 22.8441 | .0000 | 83.5456 | 14.3064 | .0000 | 31.1871 | .0000 | 87.7538 | .6460 | .9139 |
| ***Prevotellaceae*** | 6.1321 | .0000 | 16.5519 | .0000 | 76.8608 | 6.6824 | .0000 | 2.4043 | .0000 | 73.1105 | .6764 | .9139 |
| ***Veillonellaceae*** | .8237 | .0000 | 3.5178 | .0000 | 17.2915 | .2640 | .0000 | 1.1755 | .0000 | 5.7781 | .6841 | .9139 |
| ***{Unknown Class} Firmicutes*** | 1.1658 | .0000 | 3.2816 | .0000 | 15.8610 | 1.5847 | .0000 | 3.6496 | .0000 | 13.6523 | .7108 | .9139 |
| ***Lactobacillaceae*** | 17.1437 | .0000 | 31.0211 | .0000 | 94.8534 | 19.9708 | .0000 | 34.8047 | .0000 | 99.4494 | .9112 | .9982 |
| ***{Unknown Phylum} Bacteria*** | 1.2248 | .0000 | 3.2584 | .0000 | 13.6031 | 1.6482 | .0000 | 3.6039 | .0000 | 11.1970 | .9444 | .9982 |
| ***Aerococcaceae*** | .5282 | .0000 | 2.1582 | .0000 | 1.5590 | .3838 | .0000 | 1.5439 | .0000 | 7.5758 | .9870 | .9982 |
| ***Corynebacteriaceae*** | 1.7226 | .0000 | 7.6354 | .0000 | 37.5155 | .3287 | .0000 | .8709 | .0000 | 3.8961 | .9982 | .9982 |

SD - standard deviation; p-value – calculated with the non-parametric Mann–Whitney test or parametric t-test depending on the data normality assessment result; p-adj. - *p* values adjusted with Benjamini-Hochberg FDR for multiple comparisons; red font - statistically significant results (*p* < .05)

**Table S5.** The percentage abundance of the urine bacteria in the study group of BC cases and healthy controls at the genus level.

| ***Genus*** | **BC cases, n = 24** | | | | | **Controls, n = 24** | | | | | ***p-value*** | ***p-adj*.** |
| --- | --- | --- | --- | --- | --- | --- | --- | --- | --- | --- | --- | --- |
|  | **Mean** | **Median** | **SD** | **Min.** | **Max.** | **Mean** | **Median** | **SD** | **Min.** | **Max.** |  |  |
| ***Pseudomonas*** | 2,6119 | 2,4006 | 2,3761 | 0,0000 | 7,9871 | 1,4330 | 0,0183 | 2,0900 | 0,0000 | 6,6240 | 0,0257 | 0,3154 |
| ***{Unknown Order} Gammaproteobacteria*** | 10,0947 | 7,7146 | 10,2223 | 0,0000 | 38,2095 | 5,8664 | 1,5946 | 7,4902 | 0,0000 | 25,8348 | 0,0424 | 0,3154 |
| ***{Unknown Family} Enterobacterales*** | 4,2010 | 1,3059 | 7,5727 | 0,0000 | 35,9040 | 1,2249 | 0,8485 | 1,5469 | 0,0000 | 5,6381 | 0,0796 | 0,3154 |
| ***Peptoniphilus*** | 0,1933 | 0,0000 | 0,5469 | 0,0000 | 2,4081 | 0,6134 | 0,0000 | 1,4979 | 0,0000 | 6,7197 | 0,0806 | 0,3154 |
| ***Pantoea*** | 7,9640 | 8,1595 | 7,5240 | 0,0000 | 23,7858 | 4,7782 | 0,3723 | 7,1788 | 0,0000 | 23,0149 | 0,0928 | 0,3154 |
| ***Cellulosimicrobium*** | 13,8537 | 10,6936 | 13,2208 | 0,0000 | 41,6390 | 12,3178 | 1,4256 | 18,7140 | 0,0000 | 52,6102 | 0,1590 | 0,4432 |
| ***{Unknown Family} Micrococcales*** | 4,1110 | 0,3287 | 6,7357 | 0,0000 | 23,1407 | 1,8084 | 0,0053 | 3,7537 | 0,0000 | 15,6400 | 0,1825 | 0,4432 |
| ***Anaerococcus*** | 0,5596 | 0,0000 | 1,4435 | 0,0000 | 6,2958 | 0,3663 | 0,0075 | 1,0615 | 0,0000 | 4,3290 | 0,3120 | 0,6631 |
| ***Streptococcus*** | 3,9769 | 0,0000 | 16,4053 | 0,0000 | 80,5069 | 2,3496 | 0,0000 | 8,5135 | 0,0000 | 40,9091 | 0,4098 | 0,7522 |
| ***Porphyromonas*** | 1,2494 | 0,0000 | 5,7635 | 0,0000 | 28,2752 | 1,5248 | 0,0000 | 4,2192 | 0,0000 | 16,1628 | 0,4425 | 0,7522 |
| ***Escherichia-Shigella*** | 6,7876 | 0,0000 | 20,2343 | 0,0000 | 83,2920 | 10,6052 | 0,0000 | 24,9746 | 0,0000 | 85,3375 | 0,7034 | 0,9982 |
| ***{Unknown Class} Firmicutes*** | 1,1658 | 0,0000 | 3,2816 | 0,0000 | 15,8610 | 1,5847 | 0,0000 | 3,6496 | 0,0000 | 13,6523 | 0,7108 | 0,9982 |
| ***Prevotella*** | 6,0122 | 0,0000 | 16,1874 | 0,0000 | 75,0809 | 6,4840 | 0,0000 | 20,0922 | 0,0000 | 73,1105 | 0,8460 | 0,9982 |
| ***Lactobacillus*** | 16,5815 | 0,0000 | 30,5311 | 0,0000 | 94,8534 | 19,1549 | 0,0000 | 33,0589 | 0,0000 | 88,1822 | 0,9288 | 0,9982 |
| ***{Unknown Phylum} Bacteria*** | 1,2248 | 0,0000 | 3,2584 | 0,0000 | 13,6031 | 1,6482 | 0,0000 | 3,6039 | 0,0000 | 11,1970 | 0,9444 | 0,9982 |
| ***Facklamia*** | 0,5152 | 0,0000 | 2,1605 | 0,0000 | 10,5590 | 0,3822 | 0,0000 | 1,5443 | 0,0000 | 7,5758 | 0,9969 | 0,9982 |
| ***Corynebacterium*** | 1,7226 | 0,0000 | 7,6354 | 0,0000 | 37,5155 | 0,3287 | 0,0000 | 0,8709 | 0,0000 | 3,8961 | 0,9982 | 0,9982 |

SD - standard deviation; p-value – calculated with the non-parametric Mann–Whitney test or parametric t-test depending on the data normality assessment result; p-adj. - *p* values adjusted with Benjamini-Hochberg FDR for multiple comparisons; red font - statistically significant results (*p* < .05)

**Table S6.** The percentage abundance of the urine bacteria in the study group of BC cases and healthy controls at the species level.

| ***Species*** | **BC cases, n = 24** | | | | | **Controls, n = 24** | | | | | ***p*-value** | ***p*-adj.** |
| --- | --- | --- | --- | --- | --- | --- | --- | --- | --- | --- | --- | --- |
|  | **Mean** | **Median** | **SD** | **Min.** | **Max.** | **Mean** | **Median** | **SD** | **Min.** | **Max.** |  |  |
| ***Uncultured Pseudomonas sp*** | 2.5318 | 2.1903 | 2.3579 | .0000 | 7.9871 | 1.4330 | .0183 | 2.0900 | .0000 | 6.6240 | .0287 | .5502 |
| ***{Unknown Order} Gammaproteobacteria*** | 10.0947 | 7.7146 | 1.2223 | .0000 | 38.2095 | 5.8664 | 1.5946 | 7.4902 | .0000 | 25.8348 | .0424 | .5502 |
| ***Uncultured bacterium-04*** | .1888 | .0000 | .9247 | .0000 | 4.5301 | 3.8941 | .0000 | 11.1675 | .0000 | 49.6199 | .0773 | .5502 |
| ***{Unknown Species} Gardnerella*** | .2617 | .0000 | 1.2821 | .0000 | 6.2811 | 4.2573 | .0000 | 11.4424 | .0000 | 49.2290 | .0773 | .5502 |
| ***{Unknown Family} Enterobacterales*** | 4.2010 | 1.3059 | 7.5727 | .0000 | 35.9040 | 1.2249 | .8485 | 1.5469 | .0000 | 5.6381 | .0796 | .5502 |
| ***Uncultured bacterium-52*** | .1674 | .0000 | .5098 | .0000 | 2.4081 | .5424 | .0000 | 1.4017 | .0000 | 6.4180 | .0897 | .5502 |
| ***Prevotella bivia*** | 1.0873 | .0000 | 3.0084 | .0000 | 12.2772 | .0194 | .0000 | .0895 | .0000 | .4388 | .1011 | .5502 |
| ***Pantoea agglomerans*** | 6.6715 | 7.2669 | 6.0997 | .0000 | 2.3335 | 4.2196 | .3723 | 6.1734 | .0000 | 17.3495 | .1029 | .5502 |
| ***Lactobacillus crispatus*** | .6460 | .0000 | 3.1441 | .0000 | 15.4070 | 4.9238 | .0000 | 14.4271 | .0000 | 56.9468 | .1152 | .5502 |
| ***Propionibacterium lymphophilum*** | .0006 | .0000 | .0031 | .0000 | .0154 | .0228 | .0000 | .0596 | .0000 | .2097 | .1434 | .5502 |
| ***{Unknown Species} Peptoniphilus*** | .0010 | .0000 | .0051 | .0000 | .0248 | .0365 | .0000 | .0915 | .0000 | .3017 | .1434 | .5502 |
| ***Cellulosimicrobium cellulans LMG 16121*** | 13.8537 | 1.6936 | 13.2208 | .0000 | 41.6390 | 12.3178 | 1.4256 | 18.7140 | .0000 | 52.6102 | .1590 | .5502 |
| ***Uncultured bacterium-15*** | .0742 | .0000 | .3633 | .0000 | 1.7799 | .3445 | .0000 | 1.4791 | .0000 | 7.2674 | .1784 | .5502 |
| ***{Unknown Family} Micrococcales*** | 4.1110 | .3287 | 6.7357 | .0000 | 23.1407 | 1.8084 | .0053 | 3.7537 | .0000 | 15.6400 | .1825 | .5502 |
| ***Uncultured bacterium-33*** | .2361 | .0000 | .6842 | .0000 | 3.0274 | .5149 | .0000 | 2.5227 | .0000 | 12.3585 | .1914 | .5502 |
| ***{Unknown Species} Dialister*** | .0239 | .0000 | .0916 | .0000 | .4477 | .0837 | .0000 | .4102 | .0000 | 2.0098 | .1914 | .5502 |
| ***Uncultured Enterobacter sp*** | .9783 | .0000 | 1.5571 | .0000 | 4.9334 | .4172 | .0000 | 1.0064 | .0000 | 4.4960 | .2438 | .6597 |
| ***Uncultured bacterium-34*** | 3.5763 | .0000 | 14.6464 | .0000 | 71.7981 | 1.8745 | .0000 | 8.2416 | .0000 | 4.4762 | .3618 | .8488 |
| ***Gamma proteobacterium symbiont of Rubiconia intermedia*** | .2400 | .0000 | .4828 | .0000 | 1.6341 | .0949 | .0000 | .2646 | .0000 | 1.1693 | .4088 | .8488 |
| ***Dialister propionicifaciens*** | .0490 | .0000 | .1824 | .0000 | .8848 | .1142 | .0000 | .5351 | .0000 | 2.6239 | .4185 | .8488 |
| ***Porphyromonas somerae*** | .7052 | .0000 | 3.4076 | .0000 | 16.7019 | .6145 | .0000 | 2.3711 | .0000 | 11.3961 | .4185 | .8488 |
| ***Uncultured Firmicutes bacterium-01*** | 3.6906 | .0000 | 9.1621 | .0000 | 31.5973 | 2.4965 | .0000 | 7.5207 | .0000 | 28.5018 | .4461 | .8488 |
| ***Uncultured Enterobacteriaceae bacterium*** | .0915 | .0000 | .3218 | .0000 | 1.3919 | .0860 | .0000 | .2258 | .0000 | .8588 | .4609 | .8488 |
| ***Anaerococcus lactolyticus*** | .2147 | .0000 | .7274 | .0000 | 2.5890 | .0131 | .0000 | .0350 | .0000 | .1366 | .4829 | .8488 |
| ***Uncultured bacterium-05*** | .1815 | .0000 | .7858 | .0000 | 3.8509 | .1101 | .0000 | .4086 | .0000 | 1.9767 | .5048 | .8488 |
| ***Uncultured bacterium-77*** | .0528 | .0000 | .1637 | .0000 | .7569 | .0466 | .0000 | .1397 | .0000 | .6036 | .5048 | .8488 |
| ***Prevotella timonensis*** | 3.4435 | .0000 | 13.4610 | .0000 | 64.5631 | 5.4762 | .0000 | 16.9239 | .0000 | 6.7849 | .5143 | .8488 |
| ***{Unknown Species} Escherichia-Shigella*** | 3.7105 | .0000 | 1.5980 | .0000 | 44.3289 | 6.2560 | .0000 | 16.1078 | .0000 | 64.2447 | .5167 | .8488 |
| ***Uncultured organism-12*** | .2080 | .0000 | .6943 | .0000 | 3.0993 | .4891 | .0000 | 1.2987 | .0000 | 5.4991 | .6489 | .8598 |
| ***Uncultured bacterium-30*** | .0642 | .0000 | .2788 | .0000 | 1.3665 | .0579 | .0000 | .2649 | .0000 | 1.2987 | .6515 | .8598 |
| ***Uncultured bacterium-49*** | .0491 | .0000 | .1640 | .0000 | .7453 | .1461 | .0000 | .7066 | .0000 | 3.4632 | .6515 | .8598 |
| ***Vibrio sp. U32*** | .0183 | .0000 | .0628 | .0000 | .2536 | .0235 | .0000 | .0690 | .0000 | .2748 | .6515 | .8598 |
| ***{Unknown Genus} Enterobacteriaceae*** | .7135 | .0000 | 3.1949 | .0000 | 15.6464 | 1.8256 | .0000 | 6.9671 | .0000 | 32.8738 | .6515 | .8598 |
| ***Cronobacter sp*** | .0812 | .0000 | .3267 | .0000 | 1.5722 | .0948 | .0000 | .2966 | .0000 | 1.2385 | .6796 | .8598 |
| ***Uncultured bacterium-48*** | .2835 | .0000 | 1.1088 | .0000 | 5.2900 | .2996 | .0000 | 1.0179 | .0000 | 4.3290 | .6976 | .8598 |
| ***{Unknown Genus} Lactobacillaceae*** | .3261 | .0000 | .7769 | .0000 | 2.6667 | .3010 | .0000 | 1.0218 | .0000 | 4.8639 | .7046 | .8598 |
| ***{Unknown Class} Firmicutes*** | 1.1658 | .0000 | 3.2816 | .0000 | 15.8610 | 1.5847 | .0000 | 3.6496 | .0000 | 13.6523 | .7108 | .8598 |
| ***Anaerococcus sp. PH9*** | .0307 | .0000 | .1008 | .0000 | .4222 | .0292 | .0000 | .0849 | .0000 | .3735 | .7225 | .8598 |
| ***Uncultured bacterium-17*** | .1199 | .0000 | .3965 | .0000 | 1.7799 | .1984 | .0000 | .6728 | .0000 | 2.4735 | .7370 | .8598 |
| ***{Unknown Species} Prevotella*** | .2743 | .0000 | .9311 | .0000 | 4.2071 | .5552 | .0000 | 1.9562 | .0000 | 8.5415 | .7476 | .8598 |
| ***{Unknown Species} Lactobacillus*** | 8.0891 | .0000 | 15.5683 | .0000 | 56.0656 | 9.4556 | .0000 | 2.6530 | .0000 | 71.3827 | .8437 | .9466 |
| ***Uncultured bacterium-32*** | 3.8024 | .0000 | 9.0923 | .0000 | 32.3855 | 1.9139 | .0000 | 4.2742 | .0000 | 14.7417 | .8688 | .9516 |
| ***Uncultured bacterium-75*** | 1.4063 | .0000 | 4.8949 | .0000 | 22.8728 | 2.3490 | .0000 | 5.7086 | .0000 | 21.5144 | .9057 | .9584 |
| ***Corynebacterium sp. NML98-0116*** | .1159 | .0000 | .3828 | .0000 | 1.7252 | .0479 | .0000 | .1596 | .0000 | .6722 | .9378 | .9584 |
| ***{Unknown Phylum} Bacteria*** | 1.2248 | .0000 | 3.2584 | .0000 | 13.6031 | 1.6482 | .0000 | 3.6039 | .0000 | 11.1970 | .9444 | .9584 |
| ***{Unknown Species} Streptococcus*** | .3190 | .0000 | 1.4398 | .0000 | 7.0609 | .0291 | .0000 | .0946 | .0000 | .4329 | .9584 | .9584 |

SD - standard deviation; p-value – calculated with the non-parametric Mann–Whitney test or parametric t-test depending on the data normality assessment result; p-adj. - *p* values adjusted with Benjamini-Hochberg FDR for multiple comparisons; red font - statistically significant results (*p* < .05)

**Table S7.** The percentage abundance of the oral cavity rinses bacteria in the study group of BC cases and healthy controls at the phylum level.

| ***Phylum*** | **BC cases, n = 24** | | | | | **Controls, n = 23** | | | | | ***p*-value** | ***p*-adj.** |
| --- | --- | --- | --- | --- | --- | --- | --- | --- | --- | --- | --- | --- |
|  | **Mean** | **Median** | **SD** | **Min.** | **Max.** | **Mean** | **Median** | **SD** | **Min.** | **Max.** |  |  |
| ***Actinobacteriota*** | 5.0989 | 1.8500 | 7.5018 | .0000 | 28.3549 | 7.1680 | 4.2445 | 6.9352 | .0872 | 2.9991 | .1345 | .6404 |
| ***Bacteroidota*** | 29.3980 | 27.0985 | 23.6463 | .0000 | 78.3190 | 31.5386 | 37.8571 | 21.5519 | .0000 | 75.1918 | .5337 | .6404 |
| ***Firmicutes*** | 54.6535 | 6.0021 | 26.4005 | 1.5780 | 98.6224 | 47.7590 | 43.2311 | 23.3336 | 3.8363 | 89.0228 | .2759 | .6404 |
| ***Fusobacteriota*** | .7202 | .0868 | 1.2024 | .0000 | 4.9618 | .5192 | .0598 | 1.1082 | .0000 | 3.7736 | .5326 | .6404 |
| ***{Unknown Phylum} Bacteria*** | .8656 | .0955 | 1.9421 | .0000 | 8.6420 | .8273 | .3836 | 1.3251 | .0000 | 5.7354 | .3608 | .6404 |
| ***Proteobacteria*** | 9.1318 | 2.9030 | 11.8818 | .0000 | 44.0404 | 12.0956 | 3.9024 | 19.7933 | .0000 | 75.0242 | .7126 | .7126 |

SD - standard deviation; p-value – calculated with the non-parametric Mann–Whitney test or parametric t-test depending on the data normality assessment result; p-adj. - *p* values adjusted with Benjamini-Hochberg FDR for multiple comparisons

**Table S8.** The percentage abundance of the oral cavity rinses bacteria in the study group of BC cases and healthy controls at the class level.

| ***Class*** | **BC cases, n = 24** | | | | | **Controls, n = 23** | | | | | ***p*-value** | ***p*-adj.** |
| --- | --- | --- | --- | --- | --- | --- | --- | --- | --- | --- | --- | --- |
|  | **Mean** | **Median** | **SD** | **Min.** | **Max.** | **Mean** | **Median** | **SD** | **Min.** | **Max.** |  |  |
| ***Actinobacteria*** | 4.9915 | 1.4300 | 7.5149 | .0000 | 28.3549 | 7.1413 | 4.2445 | 6.9081 | .0872 | 2.9991 | .0909 | .6100 |
| ***Clostridia*** | .1277 | .0000 | .2477 | .0000 | .7634 | .0625 | .0000 | .1924 | .0000 | .8489 | .2390 | .6100 |
| ***{Unknown Phylum} Bacteria*** | .8656 | .0955 | 1.9421 | .0000 | 8.6420 | .8273 | .3836 | 1.3251 | .0000 | 5.7354 | .3608 | .6100 |
| ***Bacilli*** | 46.4768 | 52.1113 | 29.8110 | 1.2007 | 98.5321 | 39.6919 | 34.5912 | 28.1289 | 1.6725 | 85.0254 | .4795 | .6100 |
| ***Negativicutes*** | 8.0187 | 2.1514 | 12.4674 | .0000 | 47.1721 | 7.9718 | 3.7437 | 9.7366 | .0000 | 37.6307 | .4860 | .6100 |
| ***Fusobacteriia*** | .7202 | .0868 | 1.2024 | .0000 | 4.9618 | .5192 | .0598 | 1.1082 | .0000 | 3.7736 | .5326 | .6100 |
| ***Bacteroidia*** | 29.3980 | 27.0985 | 23.6463 | .0000 | 78.3190 | 31.5386 | 37.8571 | 21.5519 | .0000 | 75.1918 | .5337 | .6100 |
| ***Gammaproteobacteria*** | 9.1318 | 2.9030 | 11.8818 | .0000 | 44.0404 | 12.0956 | 3.9024 | 19.7933 | .0000 | 75.0242 | .7126 | .7126 |

SD - standard deviation; p-value – calculated with the non-parametric Mann–Whitney test or parametric t-test depending on the data normality assessment result; p-adj. - *p* values adjusted with Benjamini-Hochberg FDR for multiple comparisons

**Table S9.** The percentage abundance of the oral cavity rinses bacteria in the study group of BC cases and healthy controls at the order level.

| ***Order*** | **BC cases, n = 24** | | | | | **Controls, n = 23** | | | | | ***p*-value** | ***p*-adj.** |
| --- | --- | --- | --- | --- | --- | --- | --- | --- | --- | --- | --- | --- |
|  | **Mean** | **Median** | **SD** | **Min.** | **Max.** | **Mean** | **Median** | **SD** | **Min.** | **Max.** |  |  |
| ***Pseudomonadales*** | .0804 | .0000 | .2285 | .0000 | .9949 | .4716 | .0181 | 1.0382 | .0000 | 4.7170 | .0203 | .2846 |
| ***Micrococcales*** | 3.9091 | .4396 | 7.2095 | .0000 | 28.3549 | 5.3140 | 1.8549 | 6.5989 | .0000 | 2.4440 | .0752 | .5261 |
| ***{Unknown Order} Actinobacteria*** | .2068 | .0000 | .6043 | .0000 | 2.6037 | .2274 | .0000 | .5802 | .0000 | 2.7540 | .1344 | .5283 |
| ***Staphylococcales*** | .3101 | .0000 | 1.1711 | .0000 | 5.7709 | .1675 | .0633 | .2380 | .0000 | .7614 | .1509 | .5283 |
| ***Enterobacterales*** | 4.1426 | .6806 | 9.0151 | .0000 | 37.0202 | 3.3606 | 1.7437 | 3.8868 | .0000 | 12.1145 | .2851 | .6207 |
| ***{Unknown Phylum} Bacteria*** | .8656 | .0955 | 1.9421 | .0000 | 8.6420 | .8273 | .3836 | 1.3251 | .0000 | 5.7354 | .3608 | .6207 |
| ***Flavobacteriales*** | .1707 | .0000 | .4364 | .0000 | 1.7533 | .6710 | .0000 | 2.1592 | .0000 | 1.3774 | .3734 | .6207 |
| ***Burkholderiales*** | 4.3754 | .7621 | 6.5982 | .0000 | 22.0859 | 7.3930 | .3396 | 19.2913 | .0000 | 68.0309 | .4111 | .6207 |
| ***{Unknown Order} Gammaproteobacteria*** | .5334 | .0807 | 1.0427 | .0000 | 3.8765 | .8705 | .2787 | 1.6133 | .0000 | 7.1302 | .4285 | .6207 |
| ***Veillonellales-Selenomonadales*** | 8.0187 | 2.1514 | 12.4674 | .0000 | 47.1721 | 7.9718 | 3.7437 | 9.7366 | .0000 | 37.6307 | .4860 | .6207 |
| ***Lactobacillales*** | 45.7991 | 51.7757 | 29.1361 | 1.2007 | 91.8022 | 39.0999 | 33.9623 | 27.5683 | 1.4634 | 82.7411 | .4928 | .6207 |
| ***Fusobacteriales*** | .7202 | .0868 | 1.2024 | .0000 | 4.9618 | .5192 | .0598 | 1.1082 | .0000 | 3.7736 | .5326 | .6207 |
| ***Bacteroidales*** | 29.2273 | 27.0426 | 23.7812 | .0000 | 78.3190 | 3.8675 | 37.1069 | 21.4094 | .0000 | 75.1918 | .5763 | .6207 |
| ***Actinomycetales*** | .8225 | .1958 | 1.5473 | .0000 | 5.7692 | 1.0139 | .1282 | 2.1396 | .0000 | 9.2683 | .7877 | .7877 |

SD - standard deviation; p-value – calculated with the non-parametric Mann–Whitney test or parametric t-test depending on the data normality assessment result; p-adj. - *p* values adjusted with Benjamini-Hochberg FDR for multiple comparisons; red font - statistically significant results (*p* < .05)

**Table S10.** The percentage abundance of the oral cavity rinses bacteria in the study group of BC cases and healthy controls at the family level.

| ***Family*** | **BC cases, n = 24** | | | | | **Controls, n = 23** | | | | | ***p*-value** | ***p*-adj.** |
| --- | --- | --- | --- | --- | --- | --- | --- | --- | --- | --- | --- | --- |
|  | **Mean** | **Median** | **SD** | **Min.** | **Max.** | **Mean** | **Median** | **SD** | **Min.** | **Max.** |  |  |
| ***Erwiniaceae*** | .3055 | .0000 | .6125 | .0000 | 2.0080 | 1.1903 | .2958 | 2.0994 | .0000 | 8.7016 | .0092 | .1929 |
| ***Promicromonosporaceae*** | .7888 | .0000 | 2.0997 | .0000 | 9.0909 | 1.8435 | .1893 | 4.0499 | .0000 | 14.8936 | .0333 | .6657 |
| ***Pseudomonadaceae*** | .0804 | .0000 | .2285 | .0000 | .9949 | .2665 | .0000 | .4741 | .0000 | 1.8549 | .0470 | .8938 |
| ***Gemellaceae*** | .2896 | .0000 | 1.1721 | .0000 | 5.7709 | .1675 | .0633 | .2380 | .0000 | .7614 | .0696 | 1 |
| ***Micrococcaceae*** | 3.1204 | .2101 | 7.2041 | .0000 | 28.3549 | 3.4023 | 1.7139 | 4.5679 | .0000 | 14.2070 | .1111 | 1 |
| ***Carnobacteriaceae*** | 1.7776 | .2494 | 5.4745 | .0000 | 26.6779 | 4.0984 | .8315 | 9.1733 | .0000 | 38.4518 | .1276 | 1 |
| ***{Unknown Order} Actinobacteria*** | .2068 | .0000 | .6043 | .0000 | 2.6037 | .2274 | .0000 | .5802 | .0000 | 2.7540 | .1344 | 1 |
| ***Leptotrichiaceae*** | .3616 | .0000 | .6816 | .0000 | 2.8492 | .1083 | .0000 | .2883 | .0000 | 1.2579 | .1862 | 1 |
| ***{Unknown Phylum} Bacteria*** | .8656 | .0955 | 1.9421 | .0000 | 8.6420 | .8273 | .3836 | 1.3251 | .0000 | 5.7354 | .3608 | 1 |
| ***Flavobacteriaceae*** | .1707 | .0000 | .4364 | .0000 | 1.7533 | .6164 | .0000 | 1.9029 | .0000 | 9.1195 | .3734 | 1 |
| ***{Unknown Order} Gammaproteobacteria*** | .5334 | .0807 | 1.0427 | .0000 | 3.8765 | .8705 | .2787 | 1.6133 | .0000 | 7.1302 | .4285 | 1 |
| ***Veillonellaceae*** | 7.9949 | 2.1072 | 12.4593 | .0000 | 47.1721 | 7.9682 | 3.7437 | 9.7360 | .0000 | 37.6307 | .4599 | 1 |
| ***Neisseriaceae*** | 4.3397 | .7621 | 6.5819 | .0000 | 22.0859 | 7.1772 | .3396 | 18.6432 | .0000 | 67.1608 | .4669 | 1 |
| ***Prevotellaceae*** | 28.5017 | 25.6070 | 23.9421 | .0000 | 78.2504 | 29.5323 | 32.0017 | 21.6156 | .0000 | 72.5064 | .6506 | 1 |
| ***Actinomycetaceae*** | .7926 | .1958 | 1.5413 | .0000 | 5.7692 | .9123 | .0872 | 1.9840 | .0000 | 9.0592 | .7058 | 1 |
| ***Streptococcaceae*** | 38.1344 | 4.7590 | 28.3961 | .9639 | 9.3330 | 33.6121 | 28.6927 | 24.9411 | .1481 | 81.1098 | .7127 | 1 |
| ***Fusobacteriaceae*** | .3586 | .0000 | .8409 | .0000 | 4.0076 | .4110 | .0000 | .9652 | .0000 | 3.7084 | .7998 | 1 |
| ***Porphyromonadaceae*** | .7241 | .1113 | 1.1723 | .0000 | 4.7980 | 1.3094 | .2081 | 2.4262 | .0000 | 7.5472 | .8889 | 1 |
| ***{Unknown Family} Lactobacillales*** | .9772 | .6501 | 1.0126 | .0000 | 3.1746 | 1.1170 | .7519 | 1.5866 | .0000 | 6.8528 | .9241 | 1 |
| ***P5D1-392*** | .4834 | .0000 | 1.3235 | .0000 | 5.8725 | .1444 | .0000 | .2688 | .0000 | .8519 | .9431 | 1 |
| ***Pasteurellaceae*** | 3.7939 | .2547 | 9.1248 | .0000 | 37.0202 | 2.0100 | .2787 | 3.1395 | .0000 | 11.6740 | 1.0000 | 1 |

SD - standard deviation; p-value – calculated with the non-parametric Mann–Whitney test or parametric t-test depending on the data normality assessment result; p-adj. - *p* values adjusted with Benjamini-Hochberg FDR for multiple comparisons; red font - statistically significant results (*p* < .05)

**Table S11.** The percentage abundance of the oral cavity rinses bacteria in the study group of BC cases and healthy controls at the genus level.

| ***Genus*** | **BC cases, n = 24** | | | | | **Controls, n = 23** | | | | | ***p*-value** | ***p*-adj.** |
| --- | --- | --- | --- | --- | --- | --- | --- | --- | --- | --- | --- | --- |
|  | **Mean** | **Median** | **SD** | **Min.** | **Max.** | **Mean** | **Median** | **SD** | **Min.** | **Max.** |  |  |
| ***Pantoea*** | .3055 | .0000 | .6125 | .0000 | 2.0080 | 1.1903 | .2958 | 2.0994 | .0000 | 8.7016 | .0092 | .2205 |
| ***Cellulosimicrobium*** | .7888 | .0000 | 2.0997 | .0000 | 9.0909 | 1.8435 | .1893 | 4.0499 | .0000 | 14.8936 | .0333 | .3763 |
| ***Pseudomonas*** | .0804 | .0000 | .2285 | .0000 | .9949 | .2665 | .0000 | .4741 | .0000 | 1.8549 | .0470 | .3763 |
| ***Gemella*** | .2896 | .0000 | 1.1721 | .0000 | 5.7709 | .1675 | .0633 | .2380 | .0000 | .7614 | .0696 | .4174 |
| ***Rothia*** | 3.1204 | .2101 | 7.2041 | .0000 | 28.3549 | 3.4023 | 1.7139 | 4.5679 | .0000 | 14.2070 | .1111 | .4607 |
| ***Granulicatella*** | 1.7776 | .2494 | 5.4745 | .0000 | 26.6779 | 4.0984 | .8315 | 9.1733 | .0000 | 38.4518 | .1276 | .4607 |
| ***{Unknown Order} Actinobacteria*** | .2068 | .0000 | .6043 | .0000 | 2.6037 | .2274 | .0000 | .5802 | .0000 | 2.7540 | .1344 | .4607 |
| ***Prevotella*** | 2.8131 | 1.0887 | 3.8039 | .0000 | 12.6263 | 2.7282 | .6145 | 6.6275 | .0000 | 29.5597 | .2563 | .7690 |
| ***{Unknown Genus} Pasteurellaceae*** | .6405 | .0000 | 2.5798 | .0000 | 12.6263 | .3308 | .0000 | .7050 | .0000 | 2.7922 | .3067 | .8146 |
| ***{Unknown Phylum} Bacteria*** | .8656 | .0955 | 1.9421 | .0000 | 8.6420 | .8273 | .3836 | 1.3251 | .0000 | 5.7354 | .3608 | .8146 |
| ***Capnocytophaga*** | .1707 | .0000 | .4364 | .0000 | 1.7533 | .6164 | .0000 | 1.9029 | .0000 | 9.1195 | .3734 | .8146 |
| ***{Unknown Order} Gammaproteobacteria*** | .5334 | .0807 | 1.0427 | .0000 | 3.8765 | .8705 | .2787 | 1.6133 | .0000 | 7.1302 | .4285 | .8571 |
| ***Veillonella*** | 7.9033 | 1.4018 | 12.4550 | .0000 | 47.1721 | 7.8033 | 3.7437 | 9.6643 | .0000 | 37.6307 | .4728 | .8729 |
| ***Neisseria*** | 4.3257 | .7621 | 6.5911 | .0000 | 22.0859 | 7.1676 | .3396 | 18.6462 | .0000 | 67.1608 | .5699 | .9626 |
| ***Streptococcus*** | 38.0564 | 4.7590 | 28.3196 | .9639 | 9.3330 | 33.2659 | 28.6927 | 24.5366 | .1481 | 81.1098 | .6507 | .9626 |
| ***Leptotrichia*** | .1991 | .0000 | .4083 | .0000 | 1.4941 | .1083 | .0000 | .2883 | .0000 | 1.2579 | .6917 | .9626 |
| ***Actinomyces*** | .7926 | .1958 | 1.5413 | .0000 | 5.7692 | .9123 | .0872 | 1.9840 | .0000 | 9.0592 | .7058 | .9626 |
| ***Haemophilus*** | 1.9949 | .1903 | 5.1451 | .0000 | 24.3939 | 1.0915 | .2558 | 1.6329 | .0000 | 5.9471 | .7219 | .9626 |
| ***Fusobacterium*** | .3586 | .0000 | .8409 | .0000 | 4.0076 | .4110 | .0000 | .9652 | .0000 | 3.7084 | .7998 | .9825 |
| ***Prevotella_7*** | 23.6655 | 17.8324 | 21.6886 | .0000 | 66.9880 | 23.8222 | 3.5918 | 22.3966 | .0000 | 65.3453 | .8750 | .9825 |
| ***Porphyromonas*** | .7241 | .1113 | 1.1723 | .0000 | 4.7980 | 1.3094 | .2081 | 2.4262 | .0000 | 7.5472 | .8889 | .9825 |
| ***{Unknown Family} Lactobacillales*** | .9772 | .6501 | 1.0126 | .0000 | 3.1746 | 1.1170 | .7519 | 1.5866 | .0000 | 6.8528 | .9241 | .9825 |
| ***{Unknown Genus} P5D1-392*** | .4834 | .0000 | 1.3235 | .0000 | 5.8725 | .1444 | .0000 | .2688 | .0000 | .8519 | .9431 | .9825 |
| ***Alloprevotella*** | 1.9736 | .6538 | 3.3247 | .0000 | 12.9771 | 2.6611 | .1867 | 4.7078 | .0000 | 2.0000 | .9825 | .9825 |

SD - standard deviation; p-value – calculated with the non-parametric Mann–Whitney test or parametric t-test depending on the data normality assessment result; p-adj. - *p* values adjusted with Benjamini-Hochberg FDR for multiple comparisons; red font - statistically significant results (*p* < .05)

**Table S12.** The percentage abundance of the oral cavity rinses bacteria in the study group of BC cases and healthy controls at the species level.

| ***Species*** | **BC cases, n = 24** | | | | | **Controls, n = 23** | | | | | ***p*-value** | ***p*-adj.** |
| --- | --- | --- | --- | --- | --- | --- | --- | --- | --- | --- | --- | --- |
|  | **Mean** | **Median** | **SD** | **Min.** | **Max.** | **Mean** | **Median** | **SD** | **Min.** | **Max.** |  |  |
| ***Pantoea agglomerans*** | .2810 | .0000 | .5522 | .0000 | 1.9211 | 1.1389 | .2958 | 1.9135 | .0000 | 7.5201 | .0074 | .3272 |
| ***Cellulosimicrobium cellulans LMG 16121*** | .7888 | .0000 | 2.0997 | .0000 | 9.0909 | 1.8435 | .1893 | 4.0499 | .0000 | 14.8936 | .0333 | .5241 |
| ***Uncultured Pseudomonas sp*** | .0804 | .0000 | .2285 | .0000 | .9949 | .2665 | .0000 | .4741 | .0000 | 1.8549 | .0470 | .5241 |
| ***Abiotrophia para-adiacens*** | .7230 | .0000 | 3.0042 | .0000 | 14.7651 | 1.3409 | .0612 | 3.7416 | .0000 | 17.6570 | .0476 | .5241 |
| ***Prevotella intermedia*** | .0361 | .0000 | .1768 | .0000 | .8661 | 1.3591 | .0000 | 6.1516 | .0000 | 29.5597 | .0799 | .5912 |
| ***Uncultured bacterium-07*** | 1.8007 | .2101 | 4.3619 | .0000 | 16.9346 | 2.3316 | .7127 | 3.3749 | .0000 | 1.5161 | .0806 | .5912 |
| ***Uncultured bacterium-35*** | .2682 | .0000 | 1.0725 | .0000 | 5.2799 | .1386 | .0612 | .2034 | .0000 | .7401 | .1006 | .6321 |
| ***{Unknown Order} Actinobacteria*** | .2068 | .0000 | .6043 | .0000 | 2.6037 | .2274 | .0000 | .5802 | .0000 | 2.7540 | .1344 | .6857 |
| ***{Unknown Species} Rothia*** | 1.0754 | .0000 | 5.1541 | .0000 | 25.2713 | .4022 | .0000 | .9652 | .0000 | 4.3537 | .1451 | .6857 |
| ***Veillonella parvula*** | .0324 | .0000 | .1589 | .0000 | .7787 | .0438 | .0000 | .1208 | .0000 | .5258 | .1751 | .6857 |
| ***{Unknown Species} Capnocytophaga*** | .0528 | .0000 | .2587 | .0000 | 1.2674 | .0957 | .0000 | .3102 | .0000 | 1.2579 | .1751 | .6857 |
| ***{Unknown Species} Prevotella*** | .7693 | .0000 | 1.8145 | .0000 | 7.4074 | .1058 | .0000 | .3163 | .0000 | 1.4821 | .1870 | .6857 |
| ***Uncultured bacterium-31*** | .5829 | .0481 | 1.7953 | .0000 | 8.8926 | 1.9406 | .1439 | 5.8986 | .0000 | 28.2360 | .2285 | .7734 |
| ***Rothia dentocariosa ATCC 17931*** | .1934 | .0000 | .5892 | .0000 | 2.4147 | .6104 | .0000 | 1.1918 | .0000 | 4.1850 | .2640 | .8296 |
| ***Uncultured bacterium-15*** | 1.1172 | .2741 | 2.2000 | .0000 | 1.2525 | .9980 | .1058 | 2.5222 | .0000 | 11.1595 | .3193 | .8747 |
| ***{Unknown Phylum} Bacteria*** | .8656 | .0955 | 1.9421 | .0000 | 8.6420 | .8273 | .3836 | 1.3251 | .0000 | 5.7354 | .3608 | .8747 |
| ***{Unknown Genus} Streptococcaceae*** | .0780 | .0000 | .2917 | .0000 | 1.3781 | .3463 | .0000 | 1.0870 | .0000 | 5.1098 | .3718 | .8747 |
| ***{Unknown Family} Enterobacterales*** | .0432 | .0000 | .1450 | .0000 | .6175 | .1603 | .0000 | .5021 | .0000 | 2.3807 | .4076 | .8747 |
| ***{Unknown Order} Gammaproteobacteria*** | .5334 | .0807 | 1.0427 | .0000 | 3.8765 | .8705 | .2787 | 1.6133 | .0000 | 7.1302 | .4285 | .8747 |
| ***{Unknown Species} Lactobacillus*** | 1.3664 | .0000 | 6.0351 | .0000 | 29.6070 | .1007 | .0000 | .3343 | .0000 | 1.2308 | .4292 | .8747 |
| ***{Unknown Species} Veillonella*** | 3.7338 | .5901 | 6.7559 | .0000 | 3.9016 | 3.9918 | 2.2843 | 5.3863 | .0000 | 19.9303 | .4627 | .8747 |
| ***Uncultured bacterium-60*** | .3090 | .0000 | .8377 | .0000 | 4.0076 | .2210 | .0000 | .5208 | .0000 | 1.7903 | .4737 | .8747 |
| ***Uncultured bacterium-59*** | 3.3311 | .6741 | 6.1976 | .0000 | 23.6641 | 2.8821 | 1.5424 | 3.8650 | .0000 | 14.0969 | .4851 | .8747 |
| ***Uncultured bacterium-20*** | .1009 | .0000 | .2786 | .0000 | 1.2270 | .3970 | .0000 | 1.2445 | .0000 | 5.9748 | .4931 | .8747 |
| ***{Unknown Species} Neisseria*** | 2.1434 | .5866 | 3.4141 | .0000 | 1.5082 | 4.6666 | .3051 | 13.4774 | .0000 | 59.4586 | .5096 | .8747 |
| ***Uncultured bacterium-13*** | .1666 | .0000 | .3787 | .0000 | 1.3636 | .4064 | .0000 | 1.1959 | .0000 | 5.6604 | .5218 | .8747 |
| ***Uncultured bacterium-34*** | 2.1732 | 18.9795 | 15.9537 | .0000 | 54.8496 | 17.1126 | 14.6010 | 14.7571 | .1481 | 55.6008 | .5478 | .8747 |
| ***Uncultured Streptococcus sp*** | .7771 | .4790 | .9973 | .0000 | 4.2328 | .4729 | .4576 | .4175 | .0000 | 1.4067 | .5567 | .8747 |
| ***Uncultured bacterium-01*** | .4245 | .0000 | 1.0319 | .0000 | 4.8193 | .8288 | .0000 | 1.8120 | .0000 | 8.1533 | .5904 | .8958 |
| ***Uncultured organism-06*** | 1.4818 | .6381 | 2.3212 | .0000 | 9.7384 | 1.4288 | .7709 | 1.9367 | .0000 | 7.1661 | .6146 | .9013 |
| ***{Unknown Class} Firmicutes*** | .0303 | .0000 | .1105 | .0000 | .5328 | .0328 | .0000 | .0790 | .0000 | .2538 | .6538 | .9190 |
| ***{Unknown Order} Bacilli*** | .3344 | .0000 | 1.3714 | .0000 | 6.7299 | .3444 | .0000 | 1.2227 | .0000 | 5.8654 | .6754 | .9190 |
| ***Veillonella atypica*** | .1801 | .0000 | .4797 | .0000 | 2.2951 | .3081 | .0000 | .9682 | .0000 | 4.6690 | .7012 | .9190 |
| ***{Unknown Species} Prevotella_7*** | 4.5066 | 3.4885 | 4.5527 | .0000 | 13.5160 | 4.4808 | 2.1030 | 5.2529 | .0000 | 15.8542 | .7272 | .9190 |
| ***Uncultured bacterium-61*** | .0641 | .0000 | .1569 | .0000 | .5034 | .0961 | .0000 | .2730 | .0000 | 1.2579 | .7310 | .9190 |
| ***Uncultured bacterium-80*** | 1.6648 | .0955 | 4.6573 | .0000 | 22.0707 | .6975 | .0000 | 1.0911 | .0000 | 4.1850 | .7641 | .9339 |
| ***Uncultured bacterium-73*** | 2.1333 | .2325 | 3.4466 | .0000 | 11.6564 | 2.3829 | .1893 | 6.8978 | .0000 | 32.6561 | .7983 | .9456 |
| ***Uncultured bacterium-79*** | .0711 | .0000 | .2568 | .0000 | 1.2270 | .1379 | .0000 | .4422 | .0000 | 1.8868 | .8206 | .9456 |
| ***{Unknown Species} Streptococcus*** | 8.3543 | 6.0208 | 8.6663 | .0000 | 27.3701 | 9.6070 | 7.9699 | 12.1786 | .0000 | 57.5747 | .8585 | .9456 |
| ***Uncultured bacterium-16*** | 17.8542 | 11.4036 | 17.0478 | .0000 | 53.0120 | 17.9955 | 19.0056 | 17.1923 | .0000 | 48.5934 | .8749 | .9456 |
| ***{Unknown Species} Fusobacterium*** | .0412 | .0000 | .1005 | .0000 | .3535 | .1091 | .0000 | .3145 | .0000 | 1.2579 | .8811 | .9456 |
| ***{Unknown Family} Lactobacillales*** | .9772 | .6501 | 1.0126 | .0000 | 3.1746 | 1.1170 | .7519 | 1.5866 | .0000 | 6.8528 | .9241 | .9681 |
| ***Uncultured bacterium-14*** | 1.1979 | .1983 | 2.2191 | .0000 | 1.5317 | 1.5224 | .0000 | 2.6820 | .0000 | 11.6923 | .9552 | .9774 |
| ***{Unknown Species} Porphyromonas*** | .4572 | .0000 | .9481 | .0000 | 3.4343 | .7188 | .0000 | 1.5845 | .0000 | 5.2746 | .9811 | .9811 |

SD - standard deviation; *p*-value – calculated with the non-parametric Mann–Whitney test or parametric t-test depending on the data normality assessment result; *p*-adj. - *p* values adjusted with Benjamini-Hochberg FDR for multiple comparisons; red font - statistically significant results (*p* < .05)

**Table S13.** The percentage abundance of the urine fungi in the study group of BC cases and controls at the phylum. class. order. family. genus and species level.

| ***Phylum*** | **BC cases, n = 24** | | | | | **Controls, n = 24** | | | | | ***p-*value** | ***p*-adj.** |
| --- | --- | --- | --- | --- | --- | --- | --- | --- | --- | --- | --- | --- |
|  | **Mean** | **Median** | **SD** | **Min.** | **Max.** | **Mean** | **Median** | **SD** | **Min.** | **Max.** |  |  |
| ***Basidiomycota*** | .0000 | .0000 | .0000 | .0000 | .0000 | 4.6423 | .0000 | 13.5273 | .0000 | 5.8889 | .0392 | .1175 |
| ***Fungi_phy_Incertae_sedis*** | .0000 | .0000 | .0000 | .0000 | .0000 | 3.1865 | .0000 | 13.2442 | .0000 | 64.8855 | .0786 | .1179 |
| ***Ascomycota*** | 8.3333 | .0000 | 28.2330 | .0000 | 100.0000 | 17.1712 | .0000 | 34.3030 | .0000 | 100.0000 | .1666 | .1666 |
| ***Class*** | **Mean** | **Median** | **SD** | **Min.** | **Max.** | **Mean** | **Median** | **SD** | **Min.** | **Max.** | ***p-*value** | ***p*-adj.** |
| ***Agaricomycetes*** | 1.7684 | .0000 | 7.7085 | .0000 | 37.8586 | .0000 | .0000 | .0000 | .0000 | .0000 | .0392 | .2516 |
| ***Fungi_cls_Incertae_sedis*** | 3.1865 | .0000 | 13.2442 | .0000 | 64.8855 | .0000 | .0000 | .0000 | .0000 | .0000 | .0786 | .2516 |
| ***Microbotryomycetes*** | 1.1962 | .0000 | 5.5438 | .0000 | 27.1825 | .0000 | .0000 | .0000 | .0000 | .0000 | .1573 | .2516 |
| ***Saccharomycetes*** | 2.6866 | .0000 | 11.3719 | .0000 | 55.3571 | .0000 | .0000 | .0000 | .0000 | .0000 | .1573 | .2516 |
| ***Tremellomycetes*** | .6702 | .0000 | 2.7053 | .0000 | 13.0303 | .0000 | .0000 | .0000 | .0000 | .0000 | .1573 | .2516 |
| ***Dothideomycetes*** | 10.3180 | .0000 | 26.9730 | .0000 | 100.0000 | 8.3333 | .0000 | 28.2330 | .0000 | 100.0000 | .2898 | .3254 |
| ***Agaricostilbomycetes*** | .5622 | .0000 | 2.7541 | .0000 | 13.4921 | .0000 | .0000 | .0000 | .0000 | .0000 | .3254 | .3254 |
| ***Cystobasidiomycetes*** | .4453 | .0000 | 2.1815 | .0000 | 1.6870 | .0000 | .0000 | .0000 | .0000 | .0000 | .3254 | .3254 |
| ***Order*** | **Mean** | **Median** | **SD** | **Min.** | **Max.** | **Mean** | **Median** | **SD** | **Min.** | **Max.** | ***p-*value** | ***p*-adj.** |
| ***Capnodiales*** | 4.2628 | .0000 | 14.8789 | .0000 | 69.0276 | .0000 | .0000 | .0000 | .0000 | .0000 | .0192 | .2497 |
| ***Fungi_ord_Incertae_sedis*** | 3.1865 | .0000 | 13.2442 | .0000 | 64.8855 | .0000 | .0000 | .0000 | .0000 | .0000 | .0786 | .3408 |
| ***Dothideales*** | .6659 | .0000 | 3.0577 | .0000 | 14.9899 | .0000 | .0000 | .0000 | .0000 | .0000 | .1573 | .3408 |
| ***Russulales*** | 1.6056 | .0000 | 7.7231 | .0000 | 37.8586 | .0000 | .0000 | .0000 | .0000 | .0000 | .1573 | .3408 |
| ***Saccharomycetales*** | 2.6866 | .0000 | 11.3719 | .0000 | 55.3571 | .0000 | .0000 | .0000 | .0000 | .0000 | .1573 | .3408 |
| ***Sporidiobolales*** | 1.1962 | .0000 | 5.5438 | .0000 | 27.1825 | .0000 | .0000 | .0000 | .0000 | .0000 | .1573 | .3408 |
| ***Agaricostilbales*** | .5622 | .0000 | 2.7541 | .0000 | 13.4921 | .0000 | .0000 | .0000 | .0000 | .0000 | .3254 | .3525 |
| ***Cantharellales*** | .0636 | .0000 | .3116 | .0000 | 1.5267 | .0000 | .0000 | .0000 | .0000 | .0000 | .3254 | .3525 |
| ***Cystobasidiomycetes_ord_Incertae_sedis*** | .4453 | .0000 | 2.1815 | .0000 | 1.6870 | .0000 | .0000 | .0000 | .0000 | .0000 | .3254 | .3525 |
| ***Filobasidiales*** | .5429 | .0000 | 2.6598 | .0000 | 13.0303 | .0000 | .0000 | .0000 | .0000 | .0000 | .3254 | .3525 |
| ***Polyporales*** | .0992 | .0000 | .4860 | .0000 | 2.3810 | .0000 | .0000 | .0000 | .0000 | .0000 | .3254 | .3525 |
| ***Tremellales*** | .1272 | .0000 | .6233 | .0000 | 3.0534 | .0000 | .0000 | .0000 | .0000 | .0000 | .3254 | .3525 |
| ***Pleosporales*** | 5.3893 | .0000 | 17.4969 | .0000 | 8.7432 | 8.3333 | .0000 | 28.2330 | .0000 | 100.0000 | .4829 | .4829 |
| ***Family*** | **Mean** | **Median** | **SD** | **Min.** | **Max.** | **Mean** | **Median** | **SD** | **Min.** | **Max.** | ***p-*value** | ***p*-adj.** |
| ***Cladosporiaceae*** | 4.2628 | .0000 | 14.8789 | .0000 | 69.0276 | .0000 | .0000 | .0000 | .0000 | .0000 | .0192 | .3266 |
| ***Fungi_fam_Incertae_sedis*** | 3.1865 | .0000 | 13.2442 | .0000 | 64.8855 | .0000 | .0000 | .0000 | .0000 | .0000 | .0786 | .3457 |
| ***Peniophoraceae*** | 1.6056 | .0000 | 7.7231 | .0000 | 37.8586 | .0000 | .0000 | .0000 | .0000 | .0000 | .1573 | .3457 |
| ***Phaffomycetaceae*** | 2.4717 | .0000 | 1.3345 | .0000 | 5.1984 | .0000 | .0000 | .0000 | .0000 | .0000 | .1573 | .3457 |
| ***Saccotheciaceae*** | .6411 | .0000 | 3.0574 | .0000 | 14.9899 | .0000 | .0000 | .0000 | .0000 | .0000 | .1573 | .3457 |
| ***Sporidiobolaceae*** | 1.1962 | .0000 | 5.5438 | .0000 | 27.1825 | .0000 | .0000 | .0000 | .0000 | .0000 | .1573 | .3457 |
| ***Botryobasidiaceae*** | .0636 | .0000 | .3116 | .0000 | 1.5267 | .0000 | .0000 | .0000 | .0000 | .0000 | .3254 | .3457 |
| ***Bulleribasidiaceae*** | .1272 | .0000 | .6233 | .0000 | 3.0534 | .0000 | .0000 | .0000 | .0000 | .0000 | .3254 | .3457 |
| ***Debaryomycetaceae*** | .0165 | .0000 | .0810 | .0000 | .3968 | .0000 | .0000 | .0000 | .0000 | .0000 | .3254 | .3457 |
| ***Dothideales_fam_Incertae_sedis*** | .0248 | .0000 | .1215 | .0000 | .5952 | .0000 | .0000 | .0000 | .0000 | .0000 | .3254 | .3457 |
| ***Filobasidiaceae*** | .5429 | .0000 | 2.6598 | .0000 | 13.0303 | .0000 | .0000 | .0000 | .0000 | .0000 | .3254 | .3457 |
| ***Kondoaceae*** | .5622 | .0000 | 2.7541 | .0000 | 13.4921 | .0000 | .0000 | .0000 | .0000 | .0000 | .3254 | .3457 |
| ***Phaeosphaeriaceae*** | .0665 | .0000 | .3258 | .0000 | 1.5960 | .0000 | .0000 | .0000 | .0000 | .0000 | .3254 | .3457 |
| ***Polyporales_fam_Incertae_sedis*** | .0992 | .0000 | .4860 | .0000 | 2.3810 | .0000 | .0000 | .0000 | .0000 | .0000 | .3254 | .3457 |
| ***Saccharomycetales_fam_Incertae_sedis*** | .1984 | .0000 | .9720 | .0000 | 4.7619 | .0000 | .0000 | .0000 | .0000 | .0000 | .3254 | .3457 |
| ***Symmetrosporaceae*** | .4453 | .0000 | 2.1815 | .0000 | 1.6870 | .0000 | .0000 | .0000 | .0000 | .0000 | .3254 | .3457 |
| ***Pleosporaceae*** | 5.3228 | .0000 | 17.5149 | .0000 | 8.7432 | 8.3333 | .0000 | 28.2330 | .0000 | 100.0000 | .7370 | .7370 |
| ***Genus*** | **Mean** | **Median** | **SD** | **Min.** | **Max.** | **Mean** | **Median** | **SD** | **Min.** | **Max.** | ***p-*value** | ***p*-adj.** |
| ***Cladosporium*** | 4.2628 | .0000 | 14.8789 | .0000 | 69.0276 | .0000 | .0000 | .0000 | .0000 | .0000 | .0192 | .3254 |
| ***Fungi_gen_Incertae_sedis*** | 3.1865 | .0000 | 13.2442 | .0000 | 64.8855 | .0000 | .0000 | .0000 | .0000 | .0000 | .0786 | .3254 |
| ***Pleosporaceae_gen_Incertae_sedis*** | 5.1637 | .0000 | 17.4306 | .0000 | 8.7432 | .0000 | .0000 | .0000 | .0000 | .0000 | .0786 | .3254 |
| ***Stemphylium*** | .0000 | .0000 | .0000 | .0000 | .0000 | 8.3333 | .0000 | 28.2330 | .0000 | 100.0000 | .1572 | .3254 |
| ***Aureobasidium*** | .6411 | .0000 | 3.0574 | .0000 | 14.9899 | .0000 | .0000 | .0000 | .0000 | .0000 | .1573 | .3254 |
| ***Komagataella*** | 2.4717 | .0000 | 1.3345 | .0000 | 5.1984 | .0000 | .0000 | .0000 | .0000 | .0000 | .1573 | .3254 |
| ***Peniophora*** | 1.6056 | .0000 | 7.7231 | .0000 | 37.8586 | .0000 | .0000 | .0000 | .0000 | .0000 | .1573 | .3254 |
| ***Sporobolomyces*** | 1.1962 | .0000 | 5.5438 | .0000 | 27.1825 | .0000 | .0000 | .0000 | .0000 | .0000 | .1573 | .3254 |
| ***Alternaria*** | .1590 | .0000 | .7791 | .0000 | 3.8168 | .0000 | .0000 | .0000 | .0000 | .0000 | .3254 | .3254 |
| ***Botryobasidium*** | .0636 | .0000 | .3116 | .0000 | 1.5267 | .0000 | .0000 | .0000 | .0000 | .0000 | .3254 | .3254 |
| ***Candida*** | .1984 | .0000 | .9720 | .0000 | 4.7619 | .0000 | .0000 | .0000 | .0000 | .0000 | .3254 | .3254 |
| ***Debaryomyces*** | .0165 | .0000 | .0810 | .0000 | .3968 | .0000 | .0000 | .0000 | .0000 | .0000 | .3254 | .3254 |
| ***Dioszegia*** | .1272 | .0000 | .6233 | .0000 | 3.0534 | .0000 | .0000 | .0000 | .0000 | .0000 | .3254 | .3254 |
| ***Dothideales_gen_Incertae_sedis*** | .0248 | .0000 | .1215 | .0000 | .5952 | .0000 | .0000 | .0000 | .0000 | .0000 | .3254 | .3254 |
| ***Filobasidium*** | .5429 | .0000 | 2.6598 | .0000 | 13.0303 | .0000 | .0000 | .0000 | .0000 | .0000 | .3254 | .3254 |
| ***Kondoa*** | .5622 | .0000 | 2.7541 | .0000 | 13.4921 | .0000 | .0000 | .0000 | .0000 | .0000 | .3254 | .3254 |
| ***Phaeosphaeria*** | .0665 | .0000 | .3258 | .0000 | 1.5960 | .0000 | .0000 | .0000 | .0000 | .0000 | .3254 | .3254 |
| ***Polyporales_gen_Incertae_sedis*** | .0992 | .0000 | .4860 | .0000 | 2.3810 | .0000 | .0000 | .0000 | .0000 | .0000 | .3254 | .3254 |
| ***Symmetrospora*** | .4453 | .0000 | 2.1815 | .0000 | 1.6870 | .0000 | .0000 | .0000 | .0000 | .0000 | .3254 | .3254 |
| ***Species*** | **Mean** | **Median** | **SD** | **Min.** | **Max.** | **Mean** | **Median** | **SD** | **Min.** | **Max.** | ***p-*value** | ***p*-adj.** |
| ***Cladosporium*** | .0652 | .0000 | .2529 | .0000 | 1.2005 | .0000 | .0000 | .0000 | .0000 | .0000 | .1573 | .3254 |
| ***Aureobasidium_pullulans*** | .0355 | .0000 | .1205 | .0000 | .4484 | .0000 | .0000 | .0000 | .0000 | .0000 | .1573 | .3254 |
| ***Dothideales_sp*** | .0280 | .0000 | .1373 | .0000 | .6726 | .0000 | .0000 | .0000 | .0000 | .0000 | .3254 | .3254 |
| ***Phaeosphaeria_sp*** | .0051 | .0000 | .0247 | .0000 | .1212 | .0000 | .0000 | .0000 | .0000 | .0000 | .3254 | .3254 |
| ***Alternaria_prunicola*** | .0969 | .0000 | .4747 | .0000 | 2.3256 | .0000 | .0000 | .0000 | .0000 | .0000 | .3254 | .3254 |
| ***Pleosporaceae_sp*** | .0750 | .0000 | .3676 | .0000 | 1.8007 | .0000 | .0000 | .0000 | .0000 | .0000 | .3254 | .3254 |
| ***Stemphylium_vesicarium*** | .0000 | .0000 | .0000 | .0000 | .0000 | .0678 | .0000 | .3321 | .0000 | 1.6271 | .3254 | .3254 |
| ***Debaryomyces_udenii*** | .0187 | .0000 | .0915 | .0000 | .4484 | .0000 | .0000 | .0000 | .0000 | .0000 | .3254 | .3254 |
| ***Komagataella_phaffii*** | .1121 | .0000 | .5492 | .0000 | 2.6906 | .0000 | .0000 | .0000 | .0000 | .0000 | .3254 | .3254 |
| ***Candida_parapsilosis*** | .0280 | .0000 | .1373 | .0000 | .6726 | .0000 | .0000 | .0000 | .0000 | .0000 | .3254 | .3254 |
| ***Botryobasidium_subcoronatum*** | .0646 | .0000 | .3165 | .0000 | 1.5504 | .0000 | .0000 | .0000 | .0000 | .0000 | .3254 | .3254 |
| ***Polyporales_sp*** | .0280 | .0000 | .1373 | .0000 | .6726 | .0000 | .0000 | .0000 | .0000 | .0000 | .3254 | .3254 |
| ***Peniophora_pilatiana*** | .0282 | .0000 | .1379 | .0000 | .6757 | .0000 | .0000 | .0000 | .0000 | .0000 | .3254 | .3254 |
| ***Peniophora_simulans*** | .0429 | .0000 | .2103 | .0000 | 1.0303 | .0000 | .0000 | .0000 | .0000 | .0000 | .3254 | .3254 |
| ***Kondoa_lulangica*** | .0467 | .0000 | .2288 | .0000 | 1.1211 | .0000 | .0000 | .0000 | .0000 | .0000 | .3254 | .3254 |
| ***Sporobolomyces_roseus*** | .0561 | .0000 | .2746 | .0000 | 1.3453 | .0000 | .0000 | .0000 | .0000 | .0000 | .3254 | .3254 |
| ***Filobasidium_chernovii*** | .0152 | .0000 | .0742 | .0000 | .3636 | .0000 | .0000 | .0000 | .0000 | .0000 | .3254 | .3254 |
| ***Dioszegia_crocea*** | .0646 | .0000 | .3165 | .0000 | 1.5504 | .0000 | .0000 | .0000 | .0000 | .0000 | .3254 | .3254 |

SD - standard deviation; p-value – calculated with the non-parametric Mann–Whitney test or parametric t-test depending on the data normality assessment result; p-adj. - *p* values adjusted with Benjamini-Hochberg false-discovery rate (FDR) for multiple comparisons

**Table S14.** The percentage abundance of the oral cavity rinses fungi in the study group of BC cases and controls at the phylum. class. order. family genus and species level.

| ***Phylum*** | **BC cases, n = 24** | | | | | **Controls, n = 23** | | | | | ***p-*value** | ***p*-adj.** |
| --- | --- | --- | --- | --- | --- | --- | --- | --- | --- | --- | --- | --- |
|  | **Mean** | **Median** | **SD** | **Min.** | **Max.** | **Mean** | **Median** | **SD** | **Min.** | **Max.** |  |  |
| ***Fungi_phy_Incertae_sedis*** | .0000 | .0000 | .0000 | .0000 | .0000 | 5.0228 | .0000 | 17.5291 | .0000 | 76.1905 | .0721 | .2164 |
| ***Basidiomycota*** | .0000 | .0000 | .0000 | .0000 | .0000 | 1.7575 | .0000 | 8.0396 | .0000 | 38.5965 | .1484 | .2226 |
| ***Ascomycota*** | 2.8333 | .0000 | 41.4851 | .0000 | 100 | 1.6110 | .0000 | 28.6668 | .0000 | 100 | .6085 | .6085 |
| ***Class*** | **Mean** | **Median** | **SD** | **Min.** | **Max.** | **Mean** | **Median** | **SD** | **Min.** | **Max.** | ***p-*value** | ***p*-adj.** |
| ***Fungi_cls_Incertae_sedis*** | .0000 | .0000 | .0000 | .0000 | .0000 | 5.0228 | .0000 | 17.5291 | .0000 | 76.1905 | .0721 | .4202 |
| ***Agaricomycetes*** | .0000 | .0000 | .0000 | .0000 | .0000 | .0033 | .0000 | .0159 | .0000 | .0761 | .3152 | .4202 |
| ***Cystobasidiomycetes*** | .0000 | .0000 | .0000 | .0000 | .0000 | .0364 | .0000 | .1746 | .0000 | .8371 | .3152 | .4202 |
| ***Leotiomycetes*** | .0000 | .0000 | .0000 | .0000 | .0000 | .0165 | .0000 | .0793 | .0000 | .3805 | .3152 | .4202 |
| ***Tremellomycetes*** | .0000 | .0000 | .0000 | .0000 | .0000 | .0397 | .0000 | .1904 | .0000 | .9132 | .3152 | .4202 |
| ***Wallemiomycetes*** | .0000 | .0000 | .0000 | .0000 | .0000 | 1.6781 | .0000 | 8.0479 | .0000 | 38.5965 | .3152 | .4202 |
| ***Dothideomycetes*** | 4.1667 | .0000 | 2.4124 | .0000 | 100 | .2913 | .0000 | 1.0688 | .0000 | 4.8733 | .5744 | .6565 |
| ***Saccharomycetes*** | 16.6667 | .0000 | 38.0693 | .0000 | 100 | 14.6510 | .0000 | 33.7849 | .0000 | 100 | .8145 | .8145 |
| ***Order*** | **Mean** | **Median** | **SD** | **Min.** | **Max.** | **Mean** | **Median** | **SD** | **Min.** | **Max.** | ***p-*value** | ***p*-adj.** |
| ***Fungi_ord_Incertae_sedis*** | .0000 | .0000 | .0000 | .0000 | .0000 | 5.0228 | .0000 | 17.5291 | .0000 | 76.1905 | .0721 | .4052 |
| ***Cystobasidiomycetes_ord_Incertae_sedis*** | .0000 | .0000 | .0000 | .0000 | .0000 | .0364 | .0000 | .1746 | .0000 | .8371 | .3152 | .4052 |
| ***Cystofilobasidiales*** | .0000 | .0000 | .0000 | .0000 | .0000 | .0066 | .0000 | .0317 | .0000 | .1522 | .3152 | .4052 |
| ***Erysiphales*** | .0000 | .0000 | .0000 | .0000 | .0000 | .0165 | .0000 | .0793 | .0000 | .3805 | .3152 | .4052 |
| ***Polyporales*** | .0000 | .0000 | .0000 | .0000 | .0000 | .0033 | .0000 | .0159 | .0000 | .0761 | .3152 | .4052 |
| ***Tremellales*** | .0000 | .0000 | .0000 | .0000 | .0000 | .0331 | .0000 | .1587 | .0000 | .7610 | .3152 | .4052 |
| ***Wallemiales*** | .0000 | .0000 | .0000 | .0000 | .0000 | 1.6781 | .0000 | 8.0479 | .0000 | 38.5965 | .3152 | .4052 |
| ***Capnodiales*** | 4.1667 | .0000 | 2.4124 | .0000 | 100 | .2913 | .0000 | 1.0688 | .0000 | 4.8733 | .5744 | .6462 |
| ***Saccharomycetales*** | 16.6667 | .0000 | 38.0693 | .0000 | 100 | 14.6510 | .0000 | 33.7849 | .0000 | 10.0000 | .8145 | .8145 |
| ***Family*** | **Mean** | **Median** | **SD** | **Min.** | **Max.** | **Mean** | **Median** | **SD** | **Min.** | **Max.** | ***p-*value** | ***p*-adj.** |
| ***Fungi_fam_Incertae_sedis*** | .0000 | .0000 | .0000 | .0000 | .0000 | 5.0228 | .0000 | 17.5291 | .0000 | 76.1905 | .0721 | .4052 |
| ***Bulleraceae*** | .0000 | .0000 | .0000 | .0000 | .0000 | .0331 | .0000 | .1587 | .0000 | .7610 | .3152 | .4052 |
| ***Erysiphaceae*** | .0000 | .0000 | .0000 | .0000 | .0000 | .0165 | .0000 | .0793 | .0000 | .3805 | .3152 | .4052 |
| ***Mrakiaceae*** | .0000 | .0000 | .0000 | .0000 | .0000 | .0066 | .0000 | .0317 | .0000 | .1522 | .3152 | .4052 |
| ***Phanerochaetaceae*** | .0000 | .0000 | .0000 | .0000 | .0000 | .0033 | .0000 | .0159 | .0000 | .0761 | .3152 | .4052 |
| ***Symmetrosporaceae*** | .0000 | .0000 | .0000 | .0000 | .0000 | .0364 | .0000 | .1746 | .0000 | .8371 | .3152 | .4052 |
| ***Wallemiaceae*** | .0000 | .0000 | .0000 | .0000 | .0000 | 1.6781 | .0000 | 8.0479 | .0000 | 38.5965 | .3152 | .4052 |
| ***Cladosporiaceae*** | 4.1667 | .0000 | 2.4124 | .0000 | 100 | .2913 | .0000 | 1.0688 | .0000 | 4.8733 | .5744 | .6462 |
| ***Saccharomycetales_fam_Incertae_sedis*** | 16.6667 | .0000 | 38.0693 | .0000 | 100 | 14.6510 | .0000 | 33.7849 | .0000 | 10.0000 | .8145 | .8145 |
| ***Genus*** | **Mean** | **Median** | **SD** | **Min.** | **Max.** | **Mean** | **Median** | **SD** | **Min.** | **Max.** | ***p-*value** | ***p*-adj.** |
| ***Fungi_gen_Incertae_sedis*** | .0000 | .0000 | .0000 | .0000 | .0000 | 5.0228 | .0000 | 17.5291 | .0000 | 76.1905 | .0721 | .4052 |
| ***Bjerkandera*** | .0000 | .0000 | .0000 | .0000 | .0000 | .0033 | .0000 | .0159 | .0000 | .0761 | .3152 | .4052 |
| ***Blumeria*** | .0000 | .0000 | .0000 | .0000 | .0000 | .0165 | .0000 | .0793 | .0000 | .3805 | .3152 | .4052 |
| ***Bullera*** | .0000 | .0000 | .0000 | .0000 | .0000 | .0331 | .0000 | .1587 | .0000 | .7610 | .3152 | .4052 |
| ***Itersonilia*** | .0000 | .0000 | .0000 | .0000 | .0000 | .0066 | .0000 | .0317 | .0000 | .1522 | .3152 | .4052 |
| ***Symmetrospora*** | .0000 | .0000 | .0000 | .0000 | .0000 | .0364 | .0000 | .1746 | .0000 | .8371 | .3152 | .4052 |
| ***Wallemia*** | .0000 | .0000 | .0000 | .0000 | .0000 | 1.6781 | .0000 | 8.0479 | .0000 | 38.5965 | .3152 | .4052 |
| ***Cladosporium*** | 4.1667 | .0000 | 2.4124 | .0000 | 100 | .2913 | .0000 | 1.0688 | .0000 | 4.8733 | .5744 | .6462 |
| ***Candida*** | 16.6667 | .0000 | 38.0693 | .0000 | 100 | 14.6510 | .0000 | 33.7849 | .0000 | 10.0000 | .8145 | .8145 |
| ***Species*** | **Mean** | **Median** | **SD** | **Min.** | **Max.** | **Mean** | **Median** | **SD** | **Min.** | **Max.** | ***p-*value** | ***p*-adj.** |
| ***Candida_albicans*** | .0000 | .0000 | .0000 | .0000 | .0000 | .0407 | .0000 | .1644 | .0000 | .7797 | .1484 | .3152 |
| ***Cladosporium*** | .0000 | .0000 | .0000 | .0000 | .0000 | .0188 | .0000 | .0899 | .0000 | .4314 | .3152 | .3152 |
| ***Blumeria_graminis*** | .0000 | .0000 | .0000 | .0000 | .0000 | .0068 | .0000 | .0327 | .0000 | .1569 | .3152 | .3152 |
| ***Candida*** | .0000 | .0000 | .0000 | .0000 | .0000 | .1211 | .0000 | .5806 | .0000 | 2.7843 | .3152 | .3152 |
| ***Candida_tropicalis*** | .0000 | .0000 | .0000 | .0000 | .0000 | .0068 | .0000 | .0327 | .0000 | .1569 | .3152 | .3152 |
| ***Bjerkandera_adusta*** | .0000 | .0000 | .0000 | .0000 | .0000 | .0034 | .0000 | .0164 | .0000 | .0784 | .3152 | .3152 |
| ***Symmetrospora_oryzicola*** | .0000 | .0000 | .0000 | .0000 | .0000 | .0085 | .0000 | .0409 | .0000 | .1961 | .3152 | .3152 |
| ***Itersonilia_pannonica*** | .0000 | .0000 | .0000 | .0000 | .0000 | .0068 | .0000 | .0327 | .0000 | .1569 | .3152 | .3152 |
| ***Bullera_alba*** | .0000 | .0000 | .0000 | .0000 | .0000 | .0068 | .0000 | .0327 | .0000 | .1569 | .3152 | .3152 |
| ***Wallemia_muriae*** | .0000 | .0000 | .0000 | .0000 | .0000 | .0509 | .0000 | .2439 | .0000 | 1.1696 | .3152 | .3152 |

SD - standard deviation; p-value – calculated with the non-parametric Mann–Whitney test or parametric t-test depending on the data normality assessment result; p-adj. - *p* values adjusted with Benjamini-Hochberg false-discovery rate (FDR) for multiple comparisons;

**Table 15.** The most relevant predicted possible pathways for functional profile distinguishing between BC patients and healthy controls in urine metabolome.

| **MetaCYC ID, pathway name,**  **BC cases/HC ratio** | **Enzymes** | ***P*-value** |
| --- | --- | --- |
| **P321-PWY**  Benzoyl-CoA degradation III (anaerobic)  ↑ in BC (fold change = 1.9) | - EC 1.3.7.8, benzoyl-CoA reductase - EC 1.1.1.259, 3-hydroxypimeloyl-CoA dehydrogenase | 0.0254 |
| **PWY-6759**  Hydrogen production III  ↑ in BC (fold change = 2.2) | - EC 1.2.7.1; pyruvate:ferredoxin oxidoreductase - EC 1.2.7.6; glyceraldehyde 3-phosphate:ferredoxin oxidoreductase - EC 1.12.7.2; hydrogenase 3 | 0.0324 |
| **PWY-6785**  Hydrogen production VIII  ↑ in BC (fold change = 2.2) | - EC 1.12.7.2; hydrogenase 3 | 0.0324 |

BC- breast cancer, HC – healthy controls

**Table 16.** The most relevant predicted pathways for functional profile distinguishing between BC patients and healthy controls in oral rinses metabolome.

| **MetaCYC ID, pathway name**  **BC/HC ratio** | **Enzymes** | ***P*-value** |
| --- | --- | --- |
| **PWY-5298**  L-lysine degradation VI  ↑ in BC (fold change = 2.2) | - EC 2.6.1.68; L-lysine 6-aminotransferase - Δ1-piperideine-6-carboxylate dehydrogenase | 0.0311 |
| **M-CRESOL-DEGRADATION-PWY**  m-cresol degradation  ↑ in BC (fold change = 1.5) | - EC 1.14.13, xylenol methylhydroxylase - EC 1.1.1.90, benzyl alcohol dehydrogenase - EC 1.2.1.7, 3-hydroxy-5-methylbenzaldehyde dehydrogenase - EC 1.14.13.23, 3-hydroxybenzoate 4-hydroxylase - EC 1.14.13.24, 3-hydroxybenzoate 6-hydroxylase | 0.0361 |
| **PWY-7719**  CMP-diacetamido-8-epilegionaminic acid biosynthesis  ↓ in BC (fold change = 0.3) | - EC 4.2.1.115, UDP-N-acetylglucosamine 4,6-dehydratase (configuration-inverting) - EC 2.6.1.34, UDP-N-acetylbacillosamine transaminase - EC 2.3.1.-, UDP-4-amino-4,6-dideoxy-N-acetyl-β-L-idosamine acetyltransferase - EC 5.3.1.-, UDP-2,4-diacetamido-2,4,6-trideoxy-β-L-idopyranose C2-epimerase - EC 3.6.1.-, UDP-2,4-diacetamido-2,4,6-trideoxy-β-L-gulopyranose hydrolase - EC 2.5.1.-, diacetamido-8-epilegionaminic acid synthase - EC 1.7.7.-, diacetamido-8-epilegionaminic acid cytidylyl transferase | 0.0428 |
| **PWY-6085**  2,4-dichlorophenoxyacetate degradation  ↓ in BC (fold change = 0.2) | - EC 1.14.11, 2,4-dichlorophenoxyacetate dioxygenase - EC 1.14.13.20, 2,4-dichlorophenol 6-monooxygenase | 0.0442 |
| **PWY-6086**  4-chloro-2-methylphenoxyacetate degradation  ↓ in BC (fold change = 0.2) | - EC 1.14.11, 2,4-dichlorophenoxyacetate dioxygenase - EC 1.14.13.20, 2,4-dichlorophenol 6-monooxygenase | 0.0442 |
| **METHYLGALLATE-DEGRADATION-PWY**  methylgallate-degradation  ↓ in BC (fold change = 0.7) | - EC 1.13.11, protocatechuate 4,5-dioxygenase - EC 3.1.1.57, 2-pyrone-4,6-dicarboxylate lactonase - EC 4.2.1.83, 4-oxalomesaconate hydratase - EC 4.1.3.17, 4-hydroxy-4-methyl-2-oxoglutarate aldolase - EC 4.1.1.112, oxaloacetate decarboxylase | 0.0493 |
| **GALLATE-DEGRADATION-I-PWY**  gallate degradation II  ↓ in BC  (fold change = 0.7) | - EC 1.13.11.57, gallate dioxygenase - EC 5.3.2.8, 4-oxalomesaconate tautomerase - EC 4.2.1.83, 4-oxalomesaconate hydratase - EC 4.1.3.17, 4-hydroxy-4-methyl-2-oxoglutarate aldolase | 0.0493 |

BC- breast cancer, HC – healthy controls
